# Detection of genomic structural variations in Guizhou indigenous pigs and the comparison with other breeds

**DOI:** 10.1371/journal.pone.0194282

**Published:** 2018-03-20

**Authors:** Chang Liu, Xueqin Ran, Jiafu Wang, Sheng Li, Jianfeng Liu

**Affiliations:** 1 Institute of Agro-Bioengineering/College of Animal Science, Guizhou University, Guiyang, China; 2 Tongren University, Tongren, China; 3 College of Animal Science and Technology, China Agricultural University, Beijing, China; Northwest A&F University, CHINA

## Abstract

Genomic structural variation (SV) is noticed for the contribution to genetic diversity and phenotypic changes. Guizhou indigenous pig (GZP) has been raised for hundreds of years with many special characteristics. The present paper aimed to uncover the influence of SV on gene polymorphism and the genetic mechanisms of phenotypic traits for GZP. Eighteen GZPs were chosen for resequencing by Illumina sequencing platform. The confident SVs of GZP were called out by both programs of pindel and softSV simultaneously and compared with the SVs deduced from the genomic data of European pig (EUP) and the native pig outside of Guizhou, China (NPOG). A total of 39,166 SVs were detected and covered 27.37 Mb of pig genome. All of 76 SVs were confirmed in GZP pig population by PCR method. The SVs numbers in NPOG and GZP were about 1.8 to 1.9 times higher than that in EUP. And a SV hotspot was found out from the 20 Mb of chromosome X of GZP, which harbored 29 genes and focused on histone modification. More than half of SVs was positioned in the intergenic regions and about one third of SVs in the introns of genes. And we found that SVs tended to locate in genes produced multi-transcripts, in which a positive correlation was found out between the numbers of SV and the gene transcripts. It illustrated that the primary mode of SVs might function on the regulation of gene expression or the transcripts splicing process. A total of 1,628 protein-coding genes were disturbed by 1,956 SVs specific in GZP, in which 93 GZP-specific SV-related genes would lose their functions due to the SV interference and gathered in reproduction ability. Interestingly, the 1,628 protein-coding genes were mainly enriched in estrogen receptor binding, steroid hormone receptor binding, retinoic acid receptor binding, oxytocin signaling pathway, mTOR signaling pathway, axon guidance and cholinergic synapse pathways. It suggested that SV might be a reason for the strong adaptability and low fecundity of GZP, and 51 candidate genes would be useful for the configuration phenotype in Xiang pig breed.

## Introduction

Indigenous pig breed shows great phenotypic varieties for hair and color pattern, morphology, reproduction, growth and adaptability [1]. There present seven native pig breeds in Guizhou province, China. Some of them have borne natural and artificial selection for hundreds of years, including Xiang, Kele, Qianbei black, Guanling, Luobo pig breeds, and so on. They share preponderant features including better disease resistance, strong adaptability and favorite meat quality. But many reports show that Guizhou pigs give much lower litter sizes compared with European pig breeds [2]. For example, the average litter size is 9–11 piglets in Large White breed while only 6.6–6.9 piglets in Xiang pig and 6–8 in Kele pig [3].

It has been found that the insertion or deletion in the pivotal regions of gene did change the gene structure and expression and have a link to phenotype trait in pig. A 12-bp insertion/deletion (indel) polymorphism in exon 1 of the secreted folate binding protein (sFBP) gene is confirmed to be associated with the uterine capacity, the number of corpora lutea and the litter size in gilt [4]. An insertion in 51 bp is also found out from exon in the Testis expressed 14 (TEX14) gene and causes infertility of boar [5]. It is thought that the insertion or deletion in exon regions might affect the folding and stability of the mRNA or the translation efficiency of these genes for fecundity regulation. Additionally, a 304 bp insertion in promoter region increases the expression of the mitochondrial NAD^+^-dependent isocitrate dehydrogenase β subunit (*IDH*3*β*) gene and is correlated with a higher backfat thickness of pig [6]. Two transcripts are resulted from the insertion or deletion of 574-bp spanned exon 5 and part of 3'-UTR of dopamine D2 receptor gene [7]. Additional STAT binding site is created by a 23-bp insertion in the promoter of Toll-like receptor 5 (TLR5), which is used to recognize flagellin in the flagella of gram-positive and gram-negative bacteria [8]. Both of dopamine D2 receptor and TLR5 genes are important in pathogen susceptibility or resistance patterns in animal. PRE-1, one of SINE element specific in pig genome, is detected in the intron of vertnin gene of pig with increased numbers of vertebrae [9]. If the PRE-1 presented in the 3’-untranslated regions of the porcine prolactin receptor short form, the protein expression would be downregulated [10]. However, it is far from clear in trait regulation mechanism just focused on insertion and deletion of several genes in pig.

Structural variation (SV) in genome includes insertion, duplication, deletion, translocation and inversion with length of more than 50 bp [11–13]. It is estimated that SVs have been manifested accounting for 83.6 percent of total genetic variation [14]. Studies in human showed that SVs have been associated with schizophrenia [15], cancer [16], and complex genetic disorders [17]. Next generation sequencing (NGS) technology provides a chance for SV detection to elucidate genetic complexity and variations contributing to diverse traits on the whole genome level. Plenty of SVs were identified based on the NGS data in sheep and candidate genes functionally related to energy metabolism and body size [18]. A SV hotspot spanning 35 Mb regions on the X chromosome is identified specifically in Chinese pig by NGS [19]. Similarly, a comprehensive survey of small- and intermediate- SVs constructed a single-nucleotide resolution map in Tibetan, and contribution to pig diversity and phenotypic changes [20]. Many genetic variations might still remain under cover in other native pig breeds.

Thus, we performed whole-genome resequencing to identify SVs of five Guizhou pig breeds, including Xiang, Kele, Qianbei, Guanling, Luobo pig breeds. In addition, we download the resequencing data of other pigs from China and European and analysized simutaneously. It was looking forward to finding the specific changes in GZP genome and the relationship with their economic traits.

## Materials and methods

### Animal ethics

All animal procedures were approved by the Institutional Animal Care and Use Committee of Guizhou University and were conducted in accordance with the National Research Council Guide for the Care and Use of Laboratory Animals.

### Animal collection

Eighteen unrelated Guizhou pigs (GZPs) were utilized for resequencing. Xiang pig (XP, n = 6) was from Congjiang county, Kele pig (KL, n = 3) from Bijie county, Qianbei black pig (QB, n = 3) from Zunyi county, Guanling pig (GL, n = 3) from Anshun county, Luobo pig (LB, n = 3) from Tongren county. Blood samples were collected from the precaval vein according to standard procedures. The information for age and farm coordinate of these five breeds was shown in S1 Table.

### DNA extraction and sequencing

Genomic DNA was extracted from blood using SQ Blood DNA Kit (OMEGA, USA) and the qualified DNA was used for genome resequencing. Two paired-end libraries were constructed for each sample and the libraries were sequenced on Illumina HiSeq2500 instrument (Illumina, USA). Reference genome sequence of pig (Sscrofa 11.1) was downloaded from Ensembl (ftp://ftp.ensembl.org/pub/release-90/fasta/sus_scrofa/dna/). The raw sequencing reads was filtered by NGS QC Toolkit with default parameters [21]. Clean reads were mapped to the pig reference genome sequence using the Burrows-Wheeler Alignment software with default parameters [22]. SAMtools was used to convert the files in SAM format to BAM format [23]. Then, duplicate marking were removed using Picard package, and base quality recalibration was performed using the Genome Analysis ToolKit (GATK) program [24].

### Identification of SVs

Bioinformatics detection of genomic variation was performed on the eighteen BAM files by Pindel [25] and SoftSV softwares [26]. The default parameters were used for both programs. Since Pindel is not applicable for translocations and translocations inverted, we only chose SV types of deletions (DEL), insertions (INS), inversions (INV) and tandem duplications (DUP). Two standards were used to filter the raw data. Firstly, the short read appeared at least three paired-ends. Secondly, two softwares, pindel and softSV, were applying for SV calling and breakpoint prediction. Two SVs overlapped more than 25 bp were merged into one SV if both of SVs were belong to the same variation type at the same chromosome [27]. We only retained SV detected by both Pindel and SoftSV programs. Furthermore, to eliminate the gender effect on SV detection, data from the chromosome Y were excluded.

### SV validation

To evaluate the reliability of the data, 76 randomly selected SVs were validated using a PCR assay and direct sequencing method. SVs primer pairs for PCR were designed based on 500-bp upstream or downstream of the insertion/deletion breakpoints of the SVs based on the reference genome sequence using the primer3 algorithm (http://frodo.wi.mit.edu/primer3/) (S2 Table). The genomic DNAs were taking as /templates for validation by PCR detection. A total volume of 20 μL was used for PCR taking 1 μL of genomic DNA (80–120 ng/μL) as templates, 10 μL of 2×PCR Master Mix (Tiangen, Beijing), 0.4 μL of 0.1 μg/μL primers, and 8.2 μL of ultrapure water. The PCR program was set at 94°C for 5 min; 30 cycles of 94°C for 30 s, 60°C for 30 s and 72°C for 35 s; and a final extension of 10 min at 72°C. The PCR products were purified and then subjected to Sanger sequencing. The genotyping of six candidate genes was also performed for 284 pigs, including XP (n = 48), KL (n = 34), QB (n = 30), LB (n = 32), GL (n = 24), Large White pig (LW, n = 48), Duroc (DU, n = 24), Rongchang pig (RC, n = 44) using the same PCR approach.

### SV calling specific to Guizhou pig

To screen the specific genomic structures of Guizhou indigenous pig, we downloaded publicly available NGS data of 36 pigs from the NCBI database (https://www.ncbi.nlm.nih.gov/sra/?term=pig) (S3 Table), including eighteen native pigs outside of Guizhou (NPOG) from six breeds (Min, Rongchang, Neijiang, Tongcheng, Jinhua, Tibetan pig) in China, eighteen European pigs (EUP) from five breeds (Landrace, Large White, Hampshire, Duroc, Berkshire pig). The confident SVs of NPOG and EUP were detected using the same method and standard for GZP. Then, we applied SVmerge approach to merge SVs among different individual [28]. Only SVs detected by two or more individuals were selected into final call set. To compare the population structure, all of SVs, detected from GZP, NOPG and EUP groups, were collected for Principal Components Analysis (PCA) using the OmicShare tools online (http://www.omicshare.com/tools).

### Annotation of SV regions

The SVs were evaluated their function using the Ensembl Variant Effect Predictor tool (http://www.ensembl.org/info/docs/tools/vep/index.html). Variant annotation classified by the VEP tool as high (disruptive impact in the protein) or modifier (non-disruptive variant) severity consequences to be used in genetic analysis of phenotypic differences observed.

To test on high conserved genes overlapped with SV, we collected a set of 458 core eukaryotic genes (CEG) that exist in a wide range of species [29]. We downloaded the RefSeq peptide ID (http://korflab.ucdavis.edu/Datasets) and used the BioMart data management system (http://www.ensembl.org/biomart/martview/) to convert the RefSeq peptide ID to homologous pig Ensembl gene IDs. Then the convert results can be overlapped with our detected genes with SV. We downloaded the total pig reference genes from BioMart, a Chi squared test was performed to test whether the conserved genes regions contained less SV than the other genes.

Then, we performed Gene Ontology (GO) functional annotation and Kyoto Encyclopedia of Genes and Genomes (KEGG) pathway analysis of the genes (i.e. breed-specific or non-breed-specific with SV in GZP, and overlapping or non-overlapping with SV) using KOBAS 3.0 tool (http://kobas.cbi.pku.edu.cn/) [30,31], and P value of <0.10 determined by Fisher's exact test was set as the criteria for significance.

## Results

### Summary of sequencing and mapping

A total of 578.03 Gb raw sequences were generated by the Illumina HiSeq2500 platform. After removing the adaptor contamination and low-quality reads, we collected 545.4 Gb (94.35%) of clean sequences. On average, a pig individual obtained approximately 30.3 Gb clean reads ranged from 26.77 Gb (LB3) to 42.68 Gb (XP6). Total of 545.4 Gb clean reads were mapped to the pig reference genome assembly (Sscrofa11.1, ~2.445 Gb) using BWA software [22]. The mapped reads occupied 96.42% of the reference with an average 11.93× of sequencing depth (Table 1). In addition, we downloaded the genome data of 36 individuals originated across the world using Illumina next generation sequencing technology from NCBI database, including 18 native pigs outside of Guizhou province in China (NPOG), 18 European pigs (EUP) from five breeds. Sequencing of 36 pigs generated a total of 670.18 Gb clean reads, and the mapped read depth for 36 individuals ranged from 4.05 to 11.90 × (S3 Table).

**Table 1:** Summary of sequencing and mapping statistics.

Sample	Raw Base(G)	Clean Base(G)	Map Reads(G)	Map ratio(%)	Depth(X)	Q20(%)	GC (%)
KL1	29.51	28.64	27.31	95.36	11.17	97.66	42.77
KL2	31.20	30.32	29.21	96.34	11.95	97.75	42.62
KL3	34.76	33.59	32.51	96.78	13.30	97.4	42.44
QB1	29.94	28.85	27.91	96.74	11.42	97.39	42.06
QB2	30.72	29.69	28.75	96.83	11.76	97.38	42.30
QB3	28.90	27.81	26.72	96.08	10.93	97.33	42.19
GL1	29.20	28.01	27.15	96.93	11.10	96.56	42.82
GL2	29.42	28.49	27.59	96.84	11.28	96.95	42.95
GL3	28.38	27.33	26.51	97.00	10.84	96.73	42.99
LB1	28.86	27.39	26.47	96.64	10.83	96.76	42.96
LB2	28.10	26.97	26.15	96.96	10.70	96.83	43.05
LB3	28.23	26.77	26.02	97.20	10.64	96.61	42.72
XP1	30.11	28.31	27.42	96.86	11.21	96.30	42.52
XP2	33.52	31.69	30.63	96.66	12.53	96.50	42.26
XP3	48.46	42.25	39.82	94.25	16.29	92.90	42.42
XP4	29.22	27.41	26.56	96.90	10.87	96.50	42.64
XP5	30.88	29.20	28.22	96.64	11.54	96.19	42.94
XP6	48.62	42.68	40.23	94.26	16.45	92.75	41.73

### Identification of SVs

Two algorithms of pindel and softSV reported different numbers of SV for eighteen GZPs, with 400,779 SVs by Pindel and 453,682 SVs by softSV (S4 Table). Based on the guideline of overlap at least 25 bp at the same variation type and chromosomal coordinate [27], two or more SVs were merged, and only those SVs detected by both of pindel and softSV were retained. The confident SVs were dropped to 190,411 for GZP. Taking the same analysis strategy for GZP, the confident SVs were identified to be 56,562 SVs for EUP and 163,876 SVs for NPOG. The SV numbers of individual ranged from 970 in LA3 to 19,109 in XP3.

**Fig 1:**
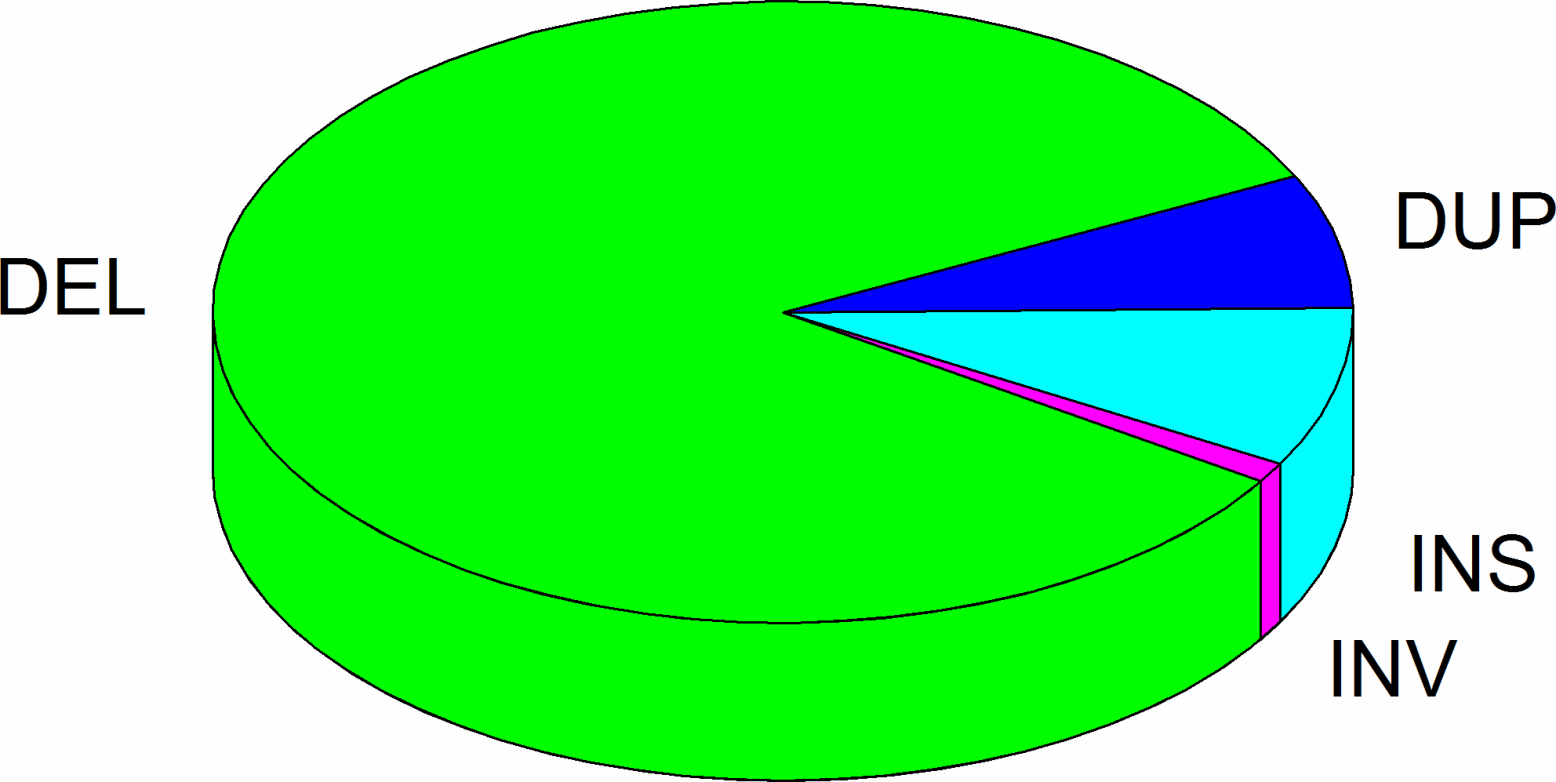
Pie chart of all types of SVs. Each slice of the pie represented one type of SV.

To get non-redundant SV, we applied SVmerge approach to merge SVs among different individual [28], in which only SVs detected by two or more individuals were selected into final call set. In total, 39,166 non-redundant SVs, named GZsv00001-GZsv39166 (S5 Table), were obtained from the 54 pig datasets, which consisted of 32,750 deletions (DEL, 83.62%,), 3,268 insertions (INS, 8.34%), 2747 tandem duplications (DUP, 7.02%), 401 inversions (INV, 1.02%) (Fig 1), the most type of identified SVs was deletion.

### SV validation

To verify the efficiency of our approach and the authenticity of the identified SVs, 76 SVs were randomly selected for validation using PCR method. The deletion or insertion genotypes of 76 SVs were confirmed (S1 Fig) and further proofed by Sanger sequencing.

### Genomic distribution of SVs

The length distribution of 39,166 SVs (Table 2) revealed that 90% DELs (29326/32750) are 50 to 1,000 bp and affected 8397.430 Kb of genome sequence, but only 3424 DELs which were larger than 1000 bp covered 12253.185 Kb of genome sequence. DELs identified in this study covered a total length near to 20.65 Mb, and the largest deletion was 16,273 bp in length. INVs and DUPs covered a total length up to 6057.886 Kb. The largest inversion and tandem duplication were 16,391 and 16,220 bp in length, respectively. The majority of inversions (44.88%) were ranged between 1 Kb and 10 Kb. However, INS identified in this study only covered a total length of 243.903 Kb, with a period from 50 to 132 bp. A total of 39,166 SVs covered 27.37 Mb pig genome.

**Table 2:** Distribution of SVs length. N: number of SV. L: SV length (bp).

Region	DEL_N	DEL_L	DUP_N	DUP_L	INS_N	INS_L	INV_N	INV_L
50–1000 bp	29326	8397430	2080	454370	3268	243903	120	58038
1–10 kb	3293	10531040	551	1912944	0	0	180	754322
10–100 kb	131	1722145	116	2367314	0	0	101	1338534

**Fig 2:**
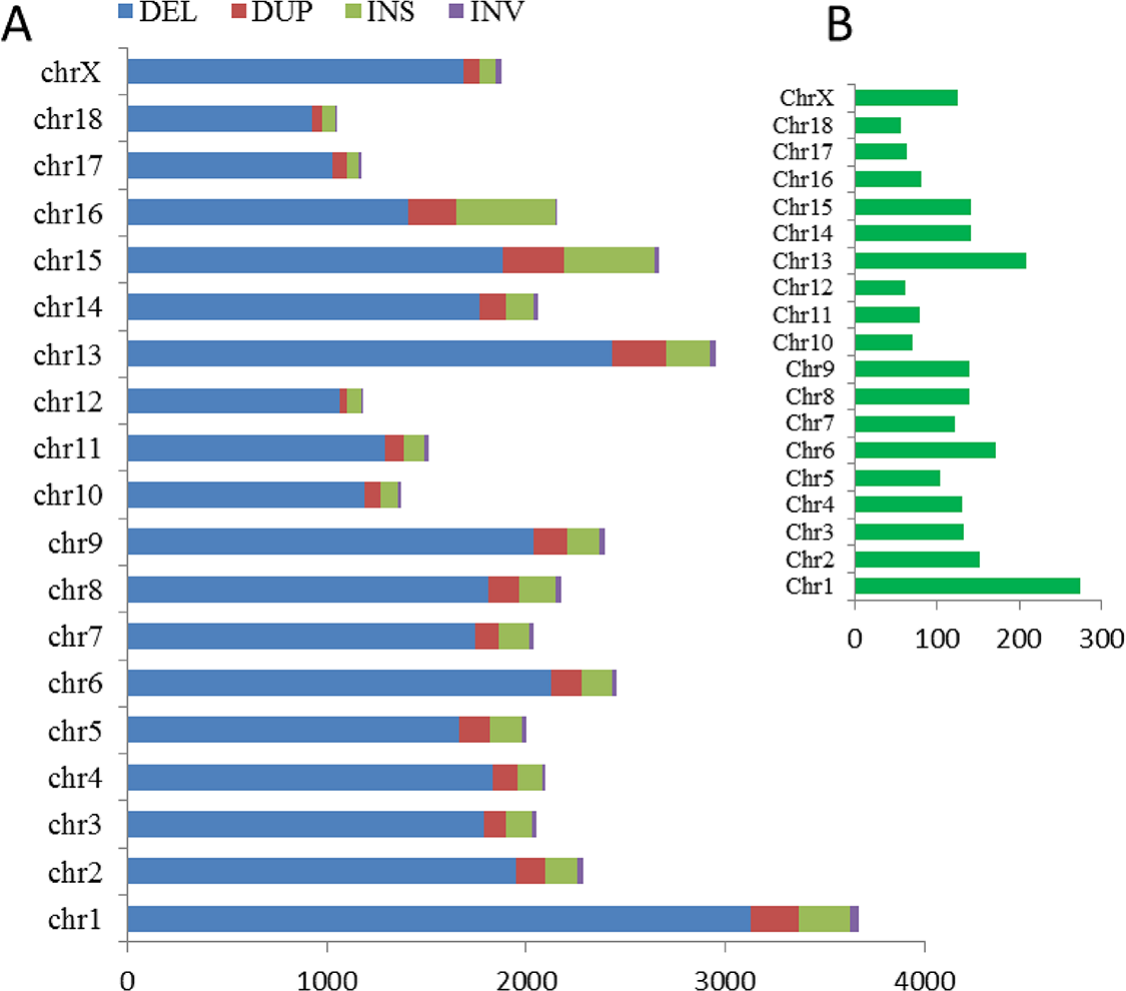
SV distribution on chromosomes. (A) Chromosomal distribution of SVs. (B) The chromosomal length of pig reference genome based on Sscrofa11.1.

**Fig 3:**
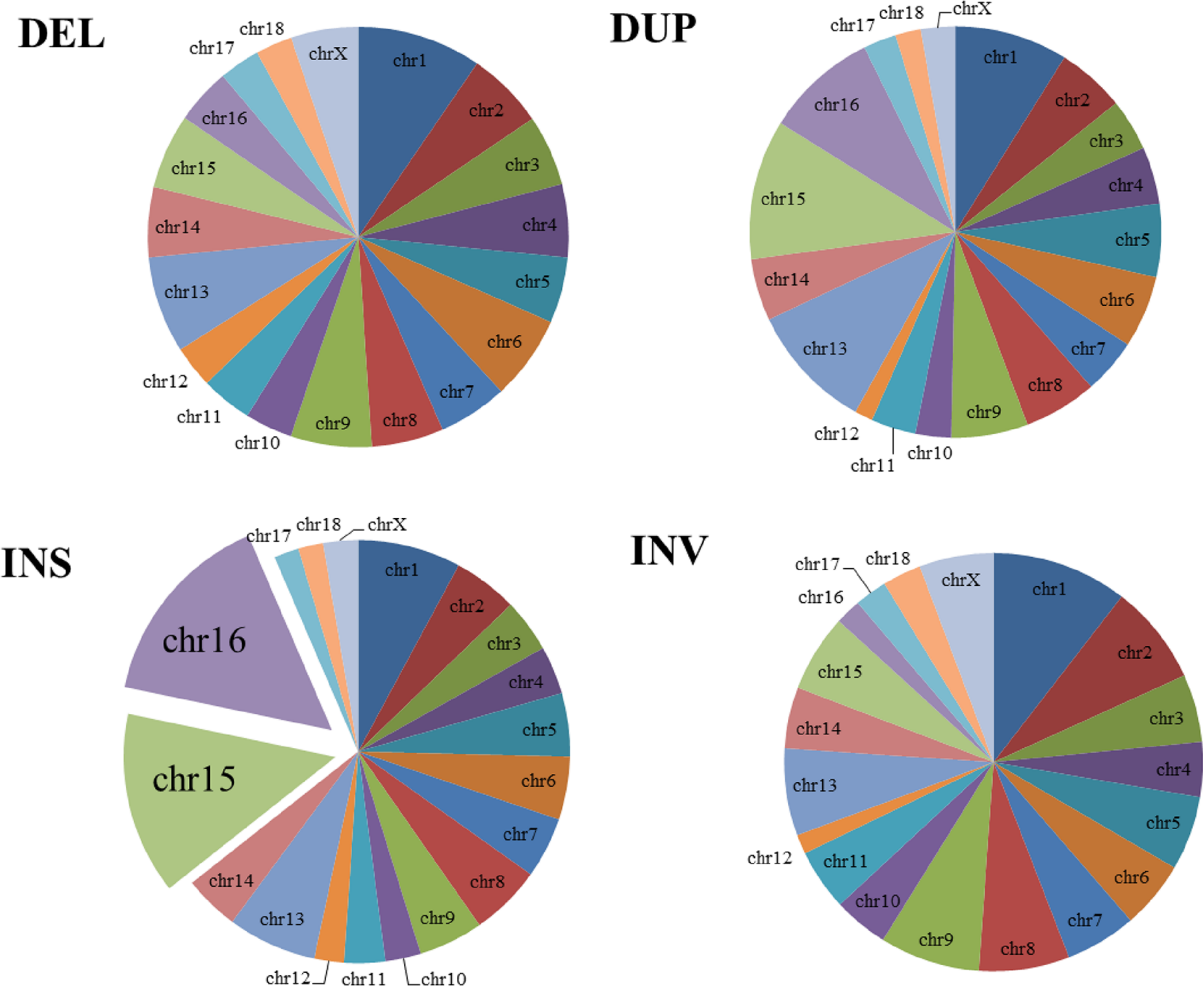
The percentage distribution of each SV type on chromosomes.

For the chromosomal distribution of SVs, the most number of SVs presented at chr1 in a ratio of 9.37%, followed by chr13 (7.54%), chr15 (6.81%), chr6 (6.28%), chr9 (6.11%), chr2 (5.84%), chr8 (5.56%), chr16 (5.51%), chr4 (5.35%), chr14 (5.25%), chr3 (5.24%), chr7 (5.219%), chr5 (5.10%), chrX (4.78%), chr11 (3.85%), chr10 (3.51%), chr12 (3.01%), chr17 (2.99%) and chr18 (2.68%) (Fig 2). Further, the chr1 contained the highest density of SVs than the others. The density of variants within each chromosome was proportional to the chromosome length, except for SVs at chr14, chrX, chr15, and chr16. Both of chr15 and chr16 were found out to give a high number of INS variants (Fig 3).

### Distribution of SV in pig population

Overall, 2,741 SVs were found only in single pig breeds, while 183 SVs presented in all sixteen pig breeds. The SV numbers were close to each other with 34,159 SVs in GZP breeds and 31,752 SVs in NPOG breeds. But it was much less in EUP breeds with 17,639 SVs (Table 3). It was noticed that the highest number of SVs was in the XP and the lowest one in the HP pig breed. In other words, the genome structure of HP is the nearest to the reference of Sscrofa 11.1, and the XP genome is the most diverse one in this study.

**Table 3:** Number of SV detected in different pig breeds.

Group	Breed	Total	DEL	DUP	INS	INV
GZP	KL	15796	14044	448	1224	80
	QB	15752	14124	434	1101	93
	GL	17260	15415	471	1291	83
	XP	28040	24352	1252	2177	259
	LB	17028	15320	479	1128	101
NPOG	NJ	13844	12658	618	517	51
	JH	12712	11870	465	335	42
	RC	15167	14088	566	455	58
	TI	17773	16340	768	600	65
	TC	17305	15875	711	645	74
	MP	19598	17733	901	819	145
EUP	DU	10998	9739	659	545	55
	LA	1983	1783	114	78	8
	LW	10822	9784	520	482	36
	HP	1559	1393	102	59	5
	BK	7644	6951	369	297	27

**Fig 4:**
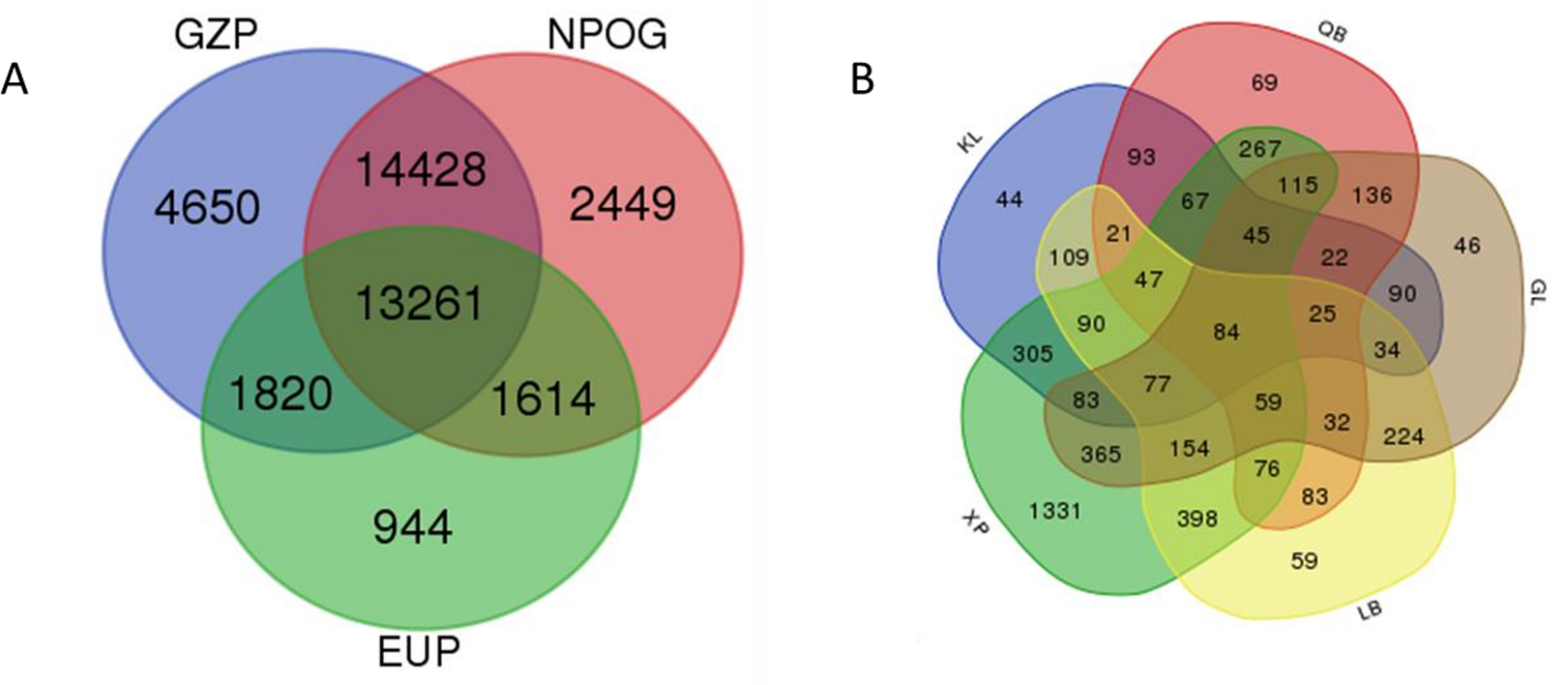
Venn diagram showing the overlap of identified SVs. **(A)** Venn diagram showing the overlap of identified SVs in the GZP, NPOG, and EUP breed. (B) Venn diagram showing the overlap of identified SVs in five GZP.

**Fig 5:**
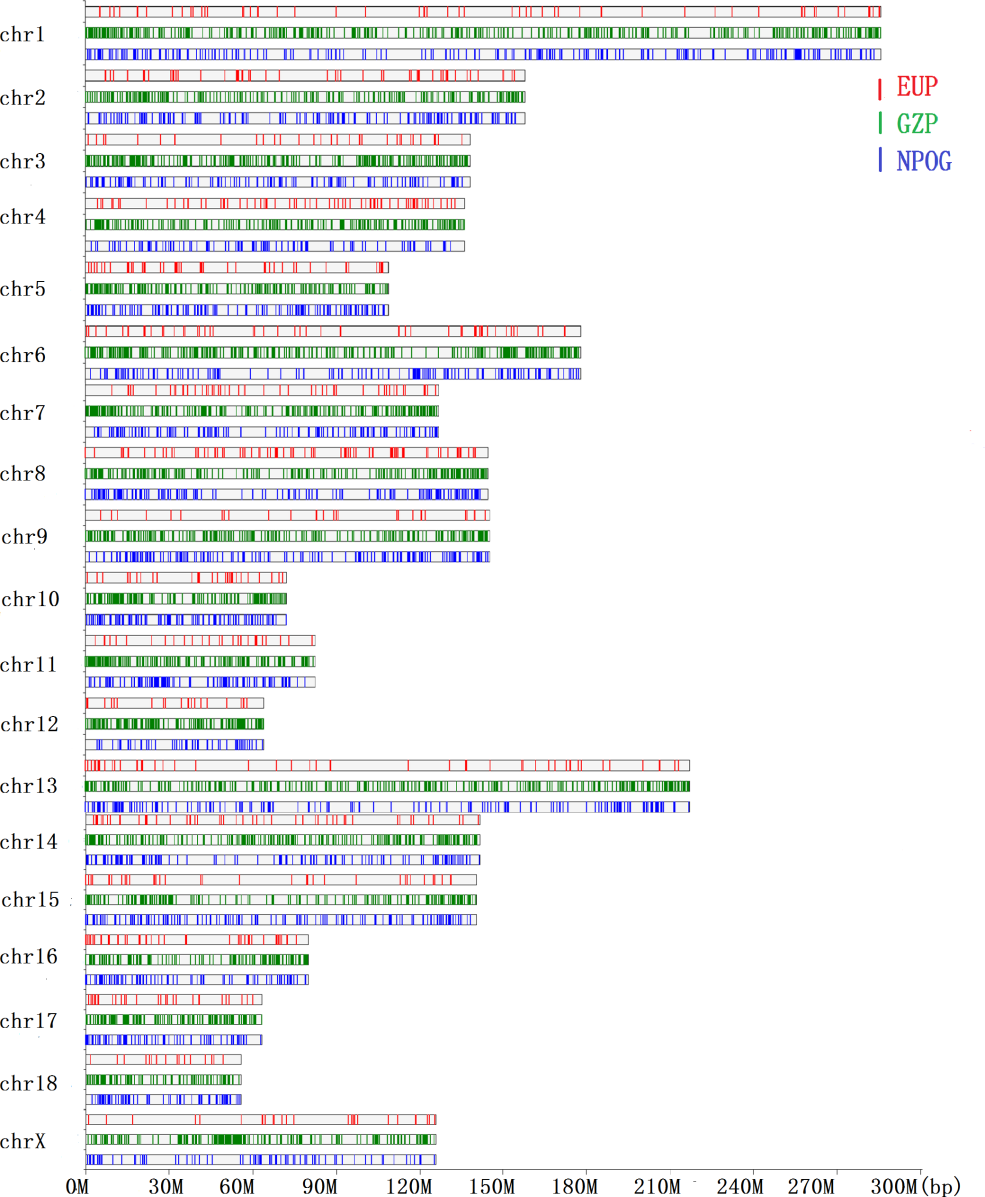
The chromosome distribution of the breed-specific in GZP, NOPG, and EUP pig breed. The vertical bars indicated different pig breed with the red, green and blue color for EUP, GZP and NPOG, respectively.

Compared SV distribution among three groups (Fig 4A), a total of 13,261 SVs were shared by three groups while the SVs with no overlap with any other breed (breed-specific) represented 4650 SVs in GZP, 2449 SVs in NPOG, and 944 SVs in EUP. SVs specific in GZP were the most abundant, while SV specific in EUP were the fewest. Of 4650 SVs specific in GZP breed, 84 SVs were common in all five breeds while the specific SV were much different in each GZP breed: 44 SVs in KL, 69 SVs in QB, 1331 SVs in XP, 46 SVs in GL and 59 SVs in LB breed (Fig 4B). The number of breed-specific SVs identified in the XP was the highest, and this finding showed that the XP breed contained plenty of variation. The distribution of these breed-specific SVs was presented in Fig 5.

**Fig 6:**
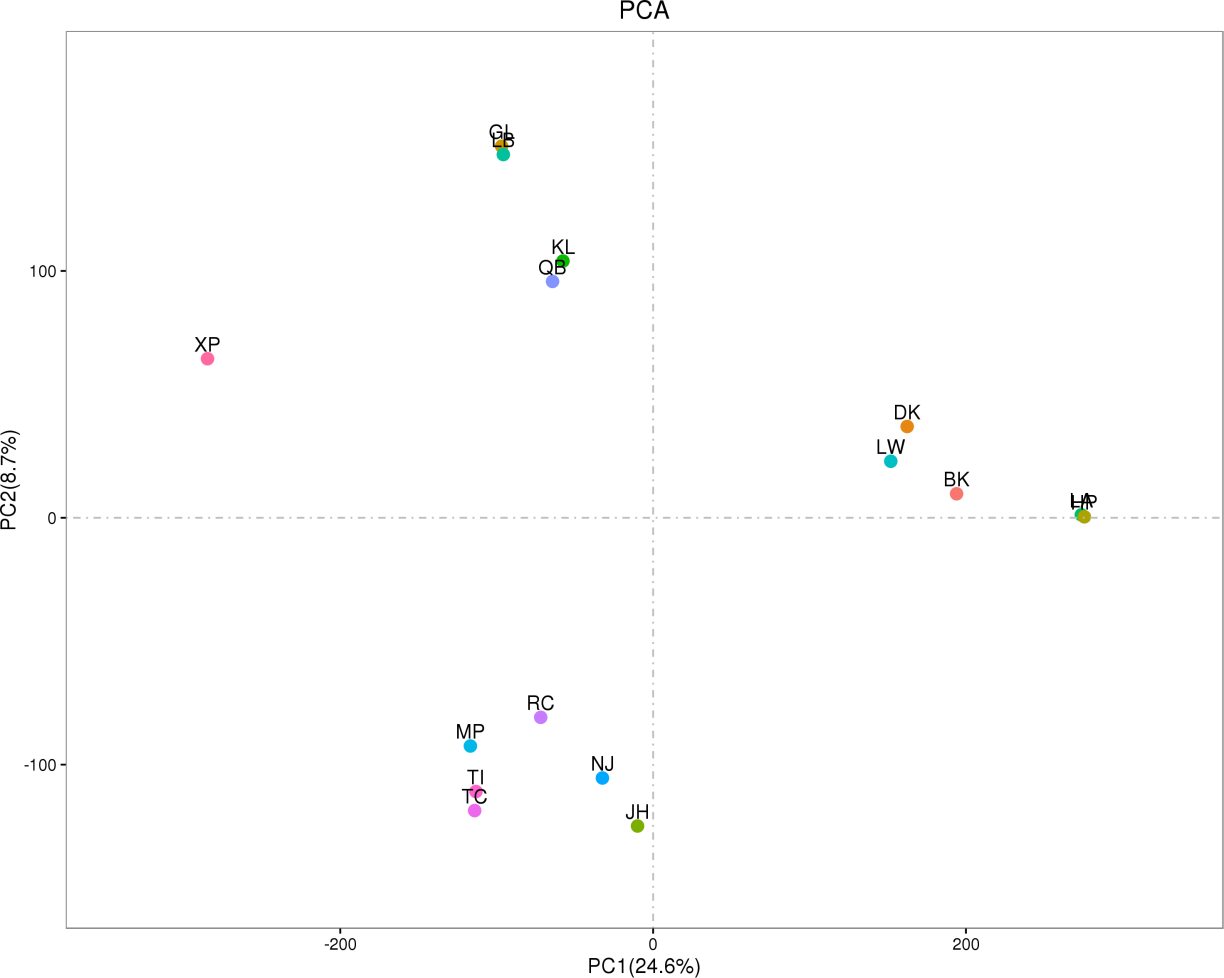
Principal component analyses for all of 16 pig breeds.

This SV pattern was clear based on by principle component analysis (PCA) analysis (Fig 6). The PC1 geographically distinguished 11 pig breeds in China from five pig breeds in Europe, whereas the PC2 captured the biological differentiation between five GZP in Guizhou and six NOPG outside of Guizhou originated from China. These findings revealed genetically distinct clusters that related to geographic locations. We also found that XP breed was detected as an outlier and had a phylogenetic distance from the other four pig breed from Guizhou province, China.

### Annotation of SVs

**Fig 7:**
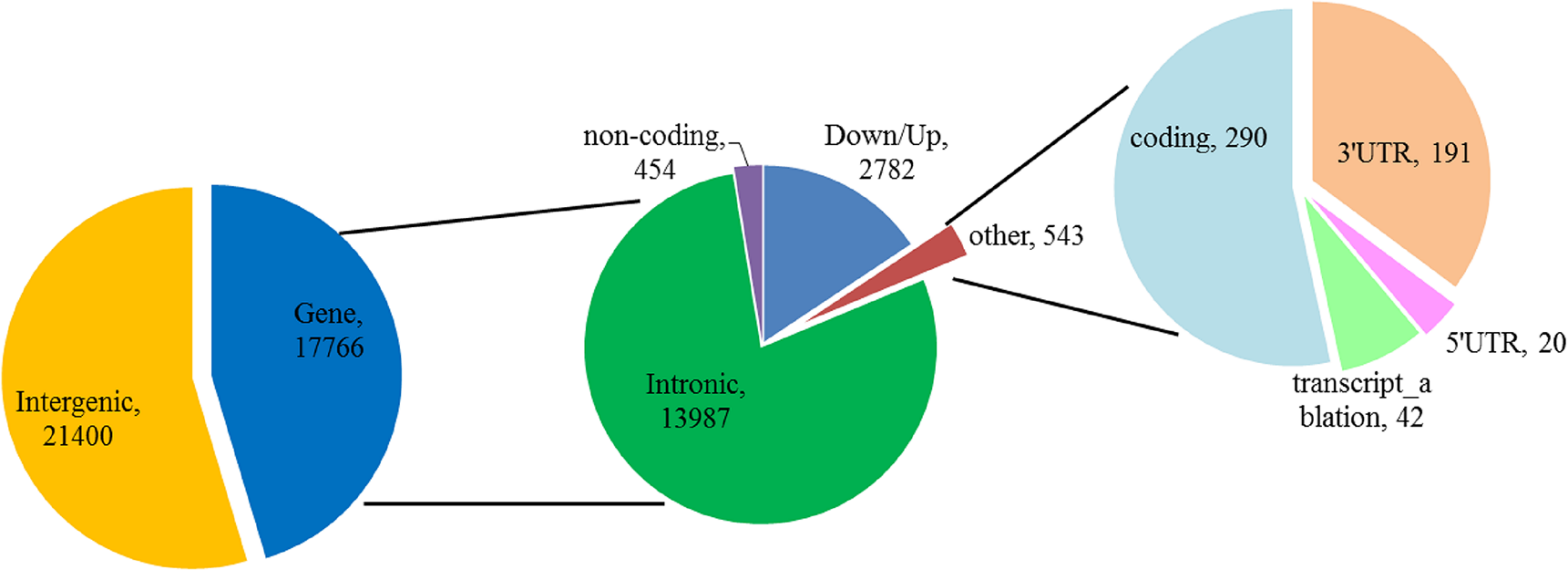
Summary of functional classification of SVs identified.

Functional annotation of the identified 39,166 SVs was performed by Variant Effect Predictor program at the Ensembl website (S5 Table). In total, majority of SVs dispersed in intergenic regions (21400/39166, 54.64%) or intronic regions (13987/39166, 35.71%). And a small number of variants were annotated in the gene of exons or untranslated flank regions (Fig 7 and S6 Table). Of the 17,766 SVs in the genic regions, the DEL was the predominant type with 15,372 SVs. Types DUP and INS ranged in the middle with 1070 and 1136 SVs, and type INV were the fewest with 188 SVs. A total of 7,881 unique Ensembl genes were overlapped or nearby to those SVs (S5 Table). Notably, most of genes contained one or two SVs (5801/7881, 73.61%). There were also a small number of genes possessed many SVs, in which both of *DIAPH*2 (diaphanous related formin 2) and *CCSER*1 (coiled-coil serine rich protein 1) contained 30 and 34 SVs, respectively. *DIAPH*2 may play a role in the development and normal function of the ovary [32]. Most of these genes contained many SVs belonged to large multigene families, including the olfactory receptor (OR), KRT, zinc finger protein, TMEM and HOXB families.

**Fig 8:**
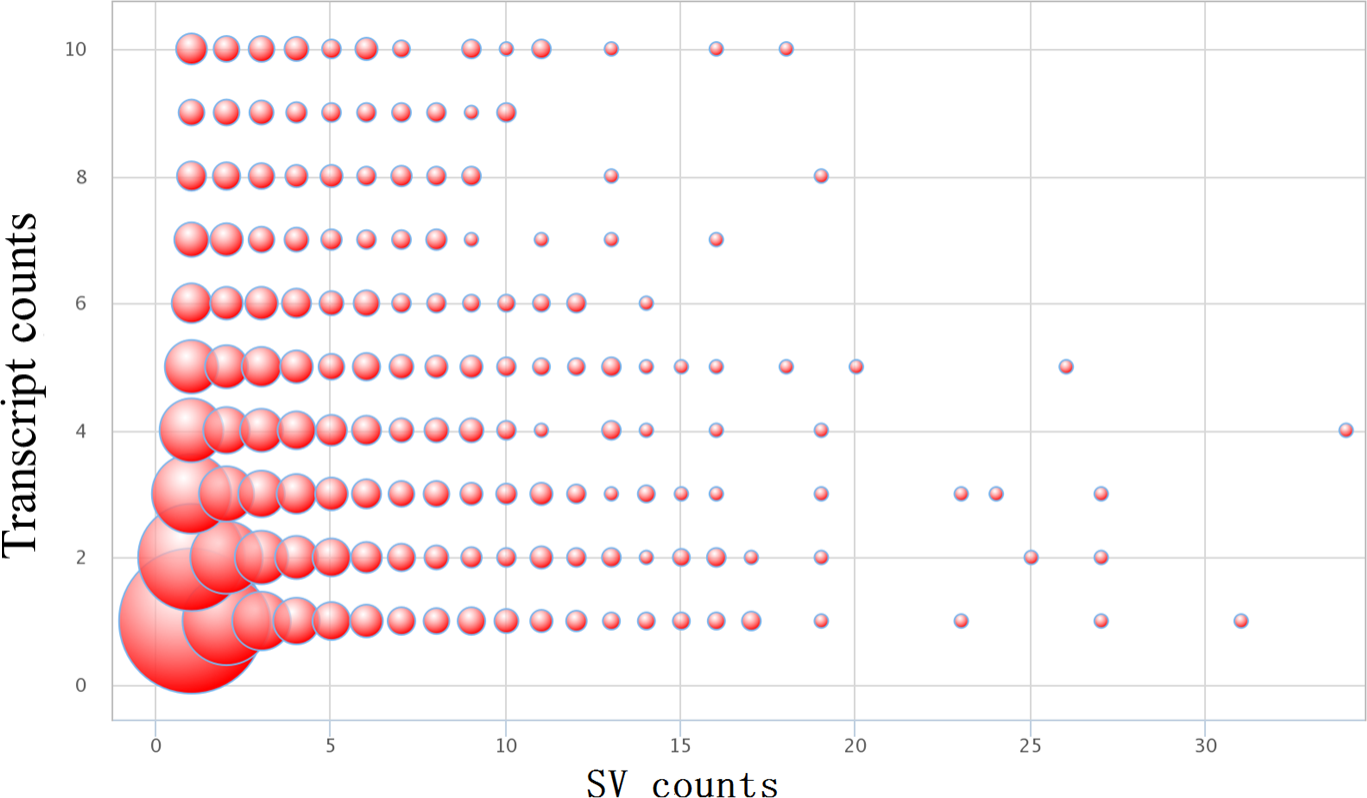
The pattern of transcripts counts changed with the SV numbers. The largest bubble showed the SV with most transcripts counts.

To the conserved eukaryotes genes, 437 unique pig genes were selected after conversion for the 458 core eukaryotic genes. About 112 core eukaryotic genes could be found out from the 7881 genes contained SV in present study. There involved 212 SVs in the 112 conserved genes. The other genes (7769) contained 17554 SVs. And conserved genes contained less SV numbers than the other genes (χ2 = 4.88, P = 0.027). Another interested finding was that SV tended to present in genes with many transcripts (Fig 8). For example, TIAM1 gene contained 18 SVs which produced ten transcripts and FARS2 gene contained 16 SVs which possessed ten transcripts. Based on STATA analysis, it showed that the SV numbers were positive correlated with the transcript numbers (Spearman = 0.231, P<0.05).

For GZP, the effect situation on gene of 34,159 SV was much similar. There were 54.04 percent (18459/34159) of SVs located in intergenic region and 15,700 SVs mapped in 7,356 Ensembl genes, including 7023 protein-coding genes, 16 pseudogenes. The remaining genes coded for 5S rRNA, snoRNA, snRNA, microRNA, lincRNA, *et al*. Of the 4,650 GZP-specific SVs, 56.10% SVs were mapped to intergenic region (2609/4650), the remaining 2041 SVs covered 1,705 Ensembl genes. For the 1,628 protein-coding genes, the ratios were 79.42% in introns, 20.15% in the regulatory regions and 0.43% in exons. Further, we identified 93 loss of function (LoF) variation from 93 protein-coding genes including 54 exon_variations of 53 genes and 39 UTR_variations of 39 genes in GZP pig breed. Of the LoF genes, we identified some interested one that might impact economic traits of GZP breed, in which five genes are involved in fertility: *CDH*5, *FTL*, *KLF*3, *BOLL*, and *ZNF*608 [33–37], and the gene *MAN*2*B*2 (Mannosidase alpha class 2B member 2) is associated with the litter size of pig [38]. We also detected *PLCL*2 gene related to immune response [39] and *LBR* (Lamin B receptor) involved in the cholesterol synthesis [40].

Furthermore, we found a SV hotspot region on the GZP chrX from 39 Mb to 59 Mb, harboring 104 SVs. In this region, 29 genes were annotated and some of them have been confirmed to associate with histone modification (S7 Table). The *TRO* (Trophinin) gene, with one frameshift variant in exon 12 due to SV (GZsv37822), encodes a membrane protein and involved in blastocyst implantation and associated with ovarian cancer [41]. *MTMR*8 (Myotubularin-related protein 8) gene was damaged by GZsv37892 for two exonic variants, which is essential for the endothelial cell differentiation and vasculature development [42]. Three affected genes, *HUWE*1, *PHF*8 and *KLF*8, are related to breast or ovary cancers [43–45]. Androgen receptor (*AR*) gene is critical for the ovarian development [46]. *ZC*3*H*12*B* gene is negative regulator in macrophage activation and may involve in host immunity and inflammatory diseases [47].

### Functional enrichment analysis for variation genes

The 1,628 genes affected by GZP-specific SVs were further used for GO and KEGG enrichment analysis (P<0.05, S8 Table). Six GO terms related with reproduction biology process were detected only from GZP but was not disturbed in both of NPOG and EUP groups. Seven genes were enriched in the six GO terms, including estrogen receptor binding (GO: 0030331), intracellular estrogen receptor signaling pathway (GO: 0030520), steroid hormone receptor binding (GO: 0035258), maternal process involved in female pregnancy (GO: 0060135), regulation of intracellular estrogen receptor signaling pathway (GO: 0033146), and retinoic acid receptor binding (GO: 0042974). Another impressed enrichment terms were associated with immunization, such as, inflammatory response to antigenic stimulus (GO: 0002437), interleukin-4 production (GO: 0032633), regulation of interleukin-1 production (GO: 0032652), T-helper 1 type immune response (GO: 0042088), macrophage activation (GO: 0042116), positive regulation of leukocyte cell-cell adhesion (GO: 1903039), positive regulation of lymphocyte activation (GO: 0051251), interleukin-1 production (GO: 0032612), positive regulation of T cell activation (GO: 0050870). Fourteen GO terms associated with adaptability, including cell projection part (GO: 0044463), axon (GO: 0030424), catecholamine transport (GO: 0051937), ATPase activity, coupled to transmembrane movement of ions, rotational mechanism (GO: 0044769), axo-dendritic transport (GO: 0008088), regulation of catecholamine secretion (GO: 0050433), cell projection cytoplasm (GO: 0032838), catecholamine secretion (GO: 0050432), dopamine transport (GO: 0015872), regulation of amine transport(GO: 0051952), dendrite (GO: 0030425), amine transport (GO: 0015837), cell projection (GO: 0042995), and positive regulation of response to biotic stimulus (GO: 0002833). Interestingly, the genes affected by SV in GZP enriched in the KEGG pathway mainly comprised metabolism and biosynthesis, reproduction, immune and adaptability, involved in oxytocin signaling pathway (ssc04921), mTOR signaling pathway (ssc04150), axon guidance (ssc04360), cholinergic synapse (ssc04725), fructose and mannose metabolism (ssc00051), glycerophospholipid metabolism (ssc00564), mucin type O-Glycan biosynthesis (ssc00512), and Glycosaminoglycan biosynthesis-heparan sulfate / heparin (ssc00534).

The seven genes involved in reproduction biology process were *ZNF*366, *LEF*1, *CNOT*1, *MED*1, *CTSB*, *HAVCR*2, and *VDR* (Table 4). Fourteen genes affected by SV in GZP enriched in the KEGG pathway of oxytocin signaling pathway, including *MEF*2*C*, *EEF*2*K*, *NFATC*3, *ROCK*1, *CD*38, *CACNA*2*D*1, *CAMKK*1, *ADCY*5, *ADCY*2, *PLCB*1, *PLCB*4, *PRKAG*2, *NOS*3 and a novel gene (Table 4). For all of 21 genes except for *CD*38, their harboured SVs located in the intron region or nearby to the gene (Table 4). These SVs might not change the coded peptides even though *CD*38 gene hold a DEL SV at the last exon, which located downstream of the stop codon.

**Table 4:** Genes affected by GZP-specific SVs enriched in GO terms and KEGG pathway related with fertility of GZP pig.

NO.	Chr	SV Start	SV End	SV Length	SV Type	Gene	Symbol	SV Location	GO/kegg ID
GZsv12513	6	20311318	20311590	272	DEL	ENSSSCG00000002799	CNOT1	Intron	GO:0030331 GO:0033146 GO:0035258 GO:0030520 GO:0042974
GZsv11540	5	78225835	78226139	304	DEL	ENSSSCG00000020864	VDR	Intron	GO:0060135 GO:0042974
GZsv18234	8	113860056	113860306	250	DEL	ENSSSCG00000009148	LEF1	Intron	GO:0030331;GO:0035258
GZsv24487	12	22838005	22838322	317	DEL	ENSSSCG00000017505	MED1	Intron	GO:0030331 GO:0033146 GO:0060135 GO:0035258 GO:0030520 GO:0042974
GZsv28563	14	15027500	15027652	152	DUP	ENSSSCG00000023666	CTSB	Intron	GO:0060135
GZsv34554	16	48808965	48809055	90	DEL	ENSSSCG00000016976	ZNF366	Intron	GO:0030331 GO:0033146 GO:0035258 GO:0030520
GZsv34801	16	66140226	66140303	77	DEL	ENSSSCG00000028875	HAVCR2	Intron	GO:0060135
GZsv04997	2	96288258	96288329	71	DEL	ENSSSCG00000014149	MEF2C	Intron	ssc04921
GZsv06434	3	23971756	23972484	730	INV	ENSSSCG00000007839	EEF2K	Intron	ssc04921
GZsv12631	6	28764371	28764445	74	DEL	ENSSSCG00000029578	NFATC3	Intron	ssc04921
GZsv13502	6	106214603	106214934	331	DEL	ENSSSCG00000021893	ROCK1	Intron	ssc04921
GZsv14469	6	165382096	165383623	1527	DEL	ENSSSCG00000003909	Novel gene*	Intron	ssc04921
GZsv16830	8	11130507	11130784	277	DEL	ENSSSCG00000008742	CD38	3’UTR	ssc04921
GZsv20391	9	98239262	98239451	189	DEL	ENSSSCG00000015402	CACNA2D1	Intron	ssc04921
GZsv24985	12	49955326	49955592	266	DEL	ENSSSCG00000017873	CAMKK1	Upstream	ssc04921
GZsv27142	13	137089754	137089859	105	INS	ENSSSCG00000027952	ADCY5	Intron	ssc04921
GZsv27144	13	137107559	137108002	443	DEL	ENSSSCG00000027952	ADCY5	Intron	ssc04921
GZsv34990	16	74352283	74352805	522	DEL	ENSSSCG00000017101	ADCY2	Intron	ssc04921
GZsv35481	17	17421770	17421871	101	INS	ENSSSCG00000007056	PLCB1	Intron	ssc04921
GZsv35498	17	18213874	18214792	918	DEL	ENSSSCG00000007058	PLCB4	Intron	ssc04921
GZsv36345	18	5484398	5484686	288	DEL	ENSSSCG00000016432	PRKAG2	Intron	ssc04921
GZsv36357	18	6232010	6232110	100	DEL	ENSSSCG00000016450	NOS3	Upstream	ssc04921

*: Uncharacterized protein.

Besides, compared between genes with SV and without SV in GZP, we found nine GO terms related with ion transport process (P<0.10, S9 Table), including ammonium transport (GO:0015696, GO:0072488, GO:0008519), lactate transport (GO:0015129, GO:0015727, GO:0035873, GO:0035879), proton-transporting two-sector ATPase complex (GO:0033178), cargo receptor activity (GO:0038024). It suggested that ammonium and lactate transports might be affected by those SVs in GZP genome by genes such as *SLC*16*A*4, *SLC*16*A*5, *SLC*16*A*7, *SLC*16*A*12, *SLC*5*A*12, *SLC*12*A*2, *SLC*22*A*2, *SLC*22*A*3, *SLC*44*A*1, *SLC*44*A*4, *RHAG* and *RHCG*. The proton-transporting ATPase included *ATP*5*A*1, *ATP*6*V*1*A*, *ATP*6*V*1*B*2 *ATP*6*V*1*C*1, *ATP*6*V*1*C*2, *ATP*6*V*1*E*1 and *ATP*6*V*1*H*.

### Identify the candidate genes associated with the body configuration in Xiang pig

Candidate genes were identified according to the following criteria: (1) genes were specific mutation only in Xiang pig (XP); (2) genes were enriched in pathways related to development and metabolism; (3) genes were associated with growth traits reported by previous studies (https://www.ncbi.nlm.nih.gov/gene/?term=growth+Sus+scrofa). The application of these criteria led to the identification of 51 candidate genes associated with body configuration in XP pig breeds, including some well-known genes such as *APOD*, *INO*80, *IGF*2*BP*3, *GSK*3*B*, *AKT*3, *MEF*2*C*, and so on (S10 Table).

**Fig 9:**
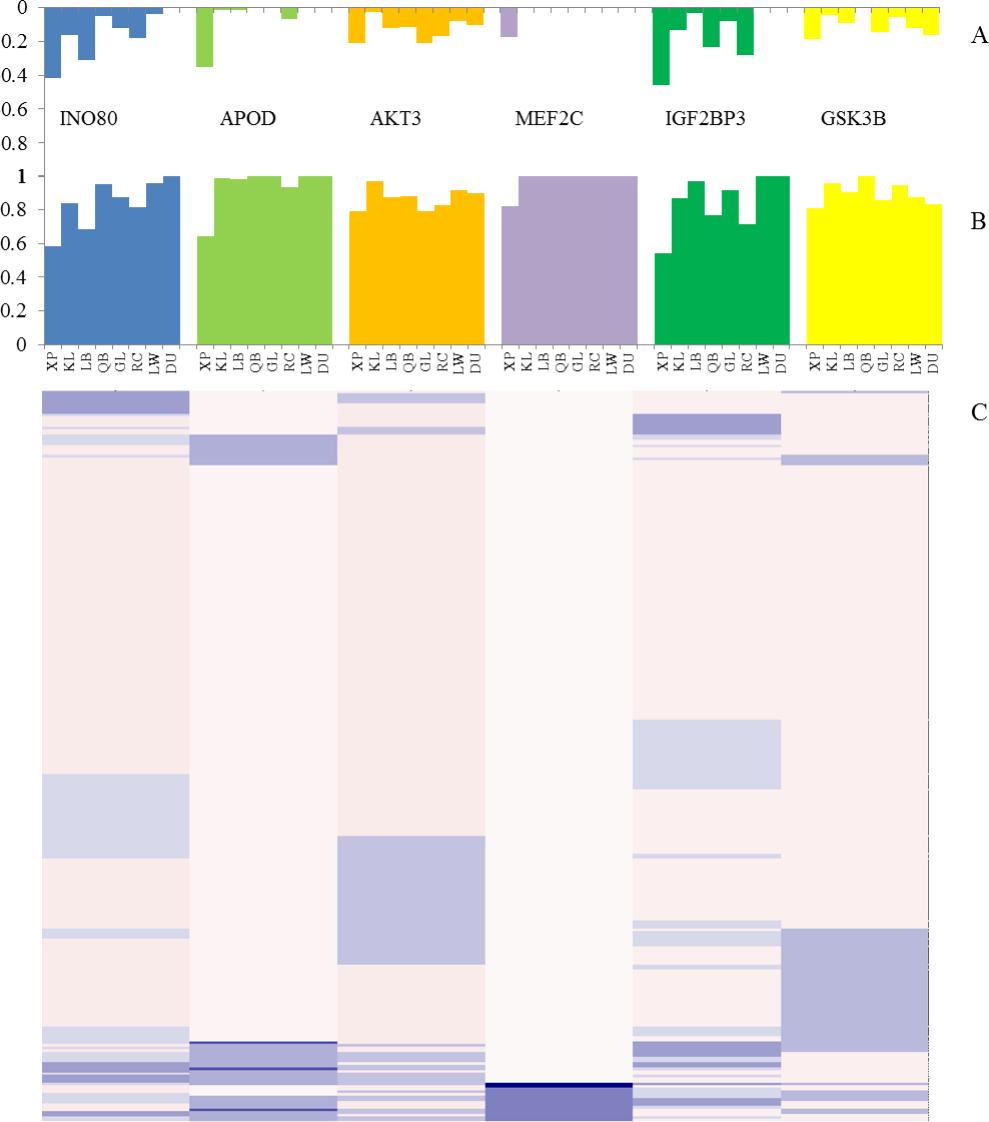
Results of Genetic population analysis. (A) Allele D frequency in pigs. (B) Allele I frequency in pigs. (C) The distribution frequencies of the six genes in 284 pigs.

To estimate the candidate genes pattern in pig population, the six candidate genes verified by PCR were genotyped using enlarged samples of 284 pigs. The genotype and allele frequencies were calculated based on the gel electrophoresis patterns. The genotype DD frequency was higher in XP than the LW, DU, KL, LB, QB, GL and RC breeds (S11 Table and Fig 9, *P* < 0.05). It indicated that the six genes could be involved in the regulation on body configuration in Xiang pig. In addition, the gene homozygosity (*Ho*), heterozygosity (*He*), polymorphism information content (*PIC*), and effective allele numbers (*Ne*) was calculated across genotype number (http://www.msrcall.com/Gdicall.aspx). The observed *He* mean was 0.605±0.096 and *PIC* mean was 0.312±0.058 in XP breed, and showed that the six markers might be informative.

## Discussion

In the present study, we performed genome resequencing for eighteen Guizhou pigs in China by NGS technology. The raw reads of GZP were filtered and combined with resequencing data from other 36 pigs of Chinese native pig outside of Guizhou and European pigs download from public database. We identified 39,166 SVs from 56 pig data, which might affected 7881 genes representing 30.45% (7881/25880) of the total genes based on the reference annotation in Sscrofa11.1. And more than 13300 SVs detected in our data were overlapped with previous SV data from thirteen Chinese and European breeds [19]. About 76 SVs were confirmed in a large of Xiang pig by PCR method.

Compared among three groups, SV numbers in GZP and NPOG were close to each other while they were much more than that in EUP. It was further confirmed by PCA results in Fig 6. Previous reports also find that Chinese indigenous pig breeds possess more plenty of genetic diversity than European breeds [19,20]. Correspondingly, the highest number of breed-specific SVs was attributed to GZP with 4,650 SV, and then NPOG with 2,449 SV, EUP with 944 SV. Inside of the five GZP, it was Xiang pig contained the highest number of breed-specific SVs (1,331) while the specific number of other four GZPs was less than a hundred. It illustrated that the genome carried many variation in Xiang pig population. It is also notable that Chinese wild boars have many variation compared with Europe pigs [19].

Notably, we identified a GZP-specific SV hotspot from 39 Mb to 59 Mb of chrX. Within the region of 20 Mb, there presented 104 SVs and contained 29 genes. Some of them have been tested for a link with reproduction including *TRO*, *MTMR*8, *HUWE*1, *PHF*8, *KLF*8, *AR* and *ZC*3*H*12*B* genes [41–47]. In addition, another work suggests a selective sweep signals region presented in chrX from 40–80 Mb of Chinese pig populations, the region involves a high *Fst* estimate between Chinese indigenous and European pig breeds [48]. It may supported that a specific SV hotspot of 39 Mb to 59 Mb on the chrX can be considered as a hotspot region to genetic diverse between Guizhou pig breed and the others.

To better understand the functions of these SV variants in GZP, we performed VEP online analysis from Ensembl. It was similar to the previous reports that most of SVs present in the intergenic region but the protein-coding region had a major influence on gene function [49]. And SV tended to distribute in genes produced multi-transcripts and showed a significant positive correlation between the SV number and the transcripts number. For example, FARS2 gene harbored 16 SVs, encodes for mitochondrial phenylalanyl-tRNA synthetase, and could generate 10 transcripts in pig. In human, a patient suffers with global developmental delay, dysarthria and tremor caused by a deletion at chromosome 6p25.1 includes all of exon 6 and parts of introns 5 and 6 of *FARS*2 [50]. It has been confirmed that intron 44 retention of the von Willebrand factor (*VWF*) gene resulting from a silent mutation in the *VWF* gene that structurally influences the splice site [51]. In our study, the *VWF* gene overlapped with six SVs in GZP (GZsv11270, GZsv11271, GZsv11272, GZsv11273, GZsv11274, and GZsv11275). We applied RegRNA 2.0 to predict the effects of SV on the changes of intron including splicing donor/acceptor sites, exon splicing enhancer (ESE), exon splicing silencer (ESS), intron splicing enhancer (ISE), and intron splicing silencer (ISS) [52]. We founded that GZsv11272, GZsv11273 and GZsv11275 contained ESE, ESS and ISE. GZsv11273 also contained a splicing acceptor site. It is reported that the splicing donor/acceptor sites may change the 3’-end of an intron or the 5’-end splice site of the intron, and lead to the production of different isoforms of transcript [53]. Transcript isoforms resulting from alternative splicing (AS) events can be viewed as having “internal-paralogs” in the same gene [54]. These “internal-paralogs” may have different functions especially in gene evolution. Taken together, SVs in the gene region might be a reason for the alternative splicing event and resulted in multi-transcripts. Beside, we found only a small parts of genes (112) contained SV from 437 CEG, which is highly conserved across eukaryotes species [29]. And these conserved genes contained less SV than the other non-conserved genes.

In addition, those genes affected by GZP-specific SVs were used for gene family annotation and function enrichment analysis by Kobas 3.0. We found seven genes (*ZNF*366, *LEF*1, *CNOT*1, *MED*1, *CTSB*, *HAVCR*2, and *VDR*) affected by SVs involved in GO terms of reproduction processes (Table 4). *ZNF*366, which encodes an evolutionarily conserved zinc finger protein, interacts with the estrogen receptor-α DNA binding domain (*ER*α DBD), represses *ER*α activity and regulating the expression of genes in response to ER [55]. *CNOT*1 contains several LXMs and interact directly in a ligand-binding domain (LBD) fashion with ERα, and represses the LBD transcriptional activation function of ERα [56]. ER mediates the function of estrogen in reproductive systems of the female and the male [57]. *LEF*1 (lymphoid enhancer-binding factor 1) has been shown to be regulated in the embryo [58] and uterus [59] during pregnancy primarily. *MED*1 (Mediator complex subunit 1) can promote nuclear hormone receptor-mediated transcription in a ligand-dependent manner [60], and regulate meiotic progression during spermatogenesis in mice [61]. *CTSB* (Cathepsins B) may modify proteins for fluid-phase transport across porcine uterine, placental, and neonatal gut epithelia [62]. *VDR* (Vitamin D3 receptor) expressed throughout central and peripheral organs of reproduction [63]. Many papers show that GZP give much lower litter sizes compared to European pig breeds with 6.6–6.9 piglets in Xiang pig, 6–8 in Kele pig, and 9–11 in Large White pig [3].It suggested that these genes affected by SV might change the reproductive performance of GZP breed.

Interestingly, the genes affected by SV in GZP enriched in several mainly pathway related to reproduction, immune and adaptability, containing oxytocin signaling pathway, mTOR signaling pathway, axon guidance and cholinergic synapse. This oxytocin signaling pathway has a role in uterine contractions during parturition and milk release during lactation [64]. The mTOR signaling pathway regulated in porcine reproductive and respiratory syndrome virus (PRRSV) infected porcine alveolar macrophages at different activation statuses [65]. The mTOR signaling pathway is also related to change synaptic plasticity in stress and depression [66], and synaptic plasticity is basic for the adaptability of the mammalian brain [67]. Axon guidance is a key stage for formation of neuronal network, and it is guided to its proper target by sensing extracellular cues in the local environment [68]. Cholinergic synapse involved in the afferent neuronal regulation of gonadotropin-releasing hormone neurons (GnRH) in rat, and GnRH is the common pathway in the hypothalamic regulation of reproduction [69]. The cholinergic system also seems likely to positively promote proliferation, differentiation, integration and affect cortical development and adult neurogenesis [70]. Therefore, these pathways might regulate reproduction, immune and adaptability, and contribution to phenotype different in GZP pig breed.

Compared the GO biological process between genes with SVs and without SVs in GZPs, there were nine ion transports processes might be affected by SVs significantly. Five involved genes were associated with the lactate transport, including *SLC*16*A*4, *SLC*16*A*12, *SLC*5*A*12, *SLC*16*A*5, and *SLC*16*A*7. For all of the five genes involved DEL variation located in the intron region. However, we retrieved 252 GO terms related with ion transports (AmiGO 2, http://amigo.geneontology.org/amigo/landing). It seemed that the effects of SV on the ion transports might be compensated by other members of SLC family.

The Xiang pig is well known Chinese miniature breed for its small body size. To identify candidate genes associated with special phenotype in Xiang pig breeds, the 51 candidate genes were collected from the integration of genes enriched in the KEGG pathway and the known genes deposited in the Gene database of NCBI. Population of 284 pigs from eight pig breeds was detected for the genotype frequencies of six genes. The deletion type of SV site, GZsv04997 in *MEF*2*C* gene, was detected only from XP breed. *MEF*2*C* (myocyte enhancer factor 2 C) is expressed in skeletal muscle and control of overall body size in mice [71]. We found that a 300 bp deletion in *APOD* gene (GZsv27094) was mainly present in XP breed, and *APOD* (Apolipoprotein D) is associated to high bone turnover, low bone mass and influences bone metabolism [72]. The remained 4 genes of genotype DD frequency were higher in XP than the LW, DU, KL, LB, QB, GL and RC breeds. *IGF*2*BP*3 (IGF2-binding protein 3) involved in transcriptional regulation of IGF2 [73] and related to bone formation [74]. *AKT*3 gene polymorphisms associated with myofiber characteristics in chickens [75]. *GSK*3*B* (glycogen synthase kinase 3 beta) plays a vital role in the muscle growth and differentiation [76–77]. *AKT*3 and *GSK*3*B* gene involved in both of thyroid hormone signaling pathway and insulin signaling pathway. It has been well-documented that the thyroid hormone contributed to the growth velocity in children with idiopathic short stature [78]. *INO*80 is required for promotion of mesenchymal stem cells (MSC) osteogenic differentiation [79]. These genes take part in the growth of muscle and bone and could be taken as a marker to phenotype characteristic in Xiang pig, but the specific effects of these genes during development and metabolism were needed further to be clear.

## Conclusion

The whole genome resequencing for five GZP breeds and comparison with data from NPOG and EUP breeds lead to identification of 39,166 SVs. This study had three highlights. Firstly, SV tended to located in the genes with multi-transcripts and the number of SV was positive correlated with that of gene transcripts. It suggested that SV might be a reason for the splice variant of pig gene. Secondly, we applied the SVs to access the population structures of these pig breeds, and got the pattern that the similarity of SV in five GZP much closer to each other than the other two groups. Thirdly, we identified 4,650 GZP-specific SVs overlapped with 1,628 protein-coding genes, in which a few SVs reshaped the coding frame of genes and about 93 genes lose function due to SV variations. Moreover, a SV hotspot was detected in 20 Mb of chrX in GZP and harbored 29 protein coding genes. The functional enrichment analysis suggested that these genes affected by GZP-specific SVs gathered in reproduction, nervous system and immune functions. Further, we identified 51 candidate genes associated with body configuration in Xiang pigs. These results provided worthwhile genomic region related to economically traits in pig and suggested that specific SVs might be a reason for the strong adaptability and low fecundity of GZP.

## Supporting information

S1 Fig Representative gel images for the confirmation of SVs in GZP pig. (PDF)

S1 Table Information of the pig breeds used for resequencing. (XLSX)

S2 Table Primers used for validation by PCR method. (XLSX)

S3 Table The information of 36 pig resequencing data downloaded from the NCBI. (XLSX)

S4 Table Counts of raw SVs in pig genomes detected by pindel and softsv programs. (XLSX)

S5 Table Confident SVs identified from sixteen pig breeds. (XLSX)

S6 Table Annotation of all SVs and the location of SVs in pig genome. (XLSX)

S7 Table The genes in the SV hotspot on chrX of GZP. (XLSX)

S8 Table KEGG Pathway analysis and Gene ontology (GO) annotation in breed-specific SVs. (XLSX)

S9 Table KEGG and GO annotations between gene with SV and gene without SV in GZP. (XLSX)

S10 Table Candidate genes associated with the body configuration in Xiang pig. (XLSX)

S11 Table The frequency of the six genes distributed in the pig populations. (XLSX)
