# Supplementary material for: Detection of genomic structural variations in Guizhou indigenous pigs and the comparison with other breeds
Source: PLoS One. 2018 Mar 20;13(3):e0194282. doi: 10.1371/journal.pone.0194282 (PMC5860705; doi:10.1371/journal.pone.0194282)
Supplement: S1 Fig — (PDF) [file pone.0194282.s001.pdf]

S1 Fig. Representative gel images for the confirmation of SVs in GZP pig.

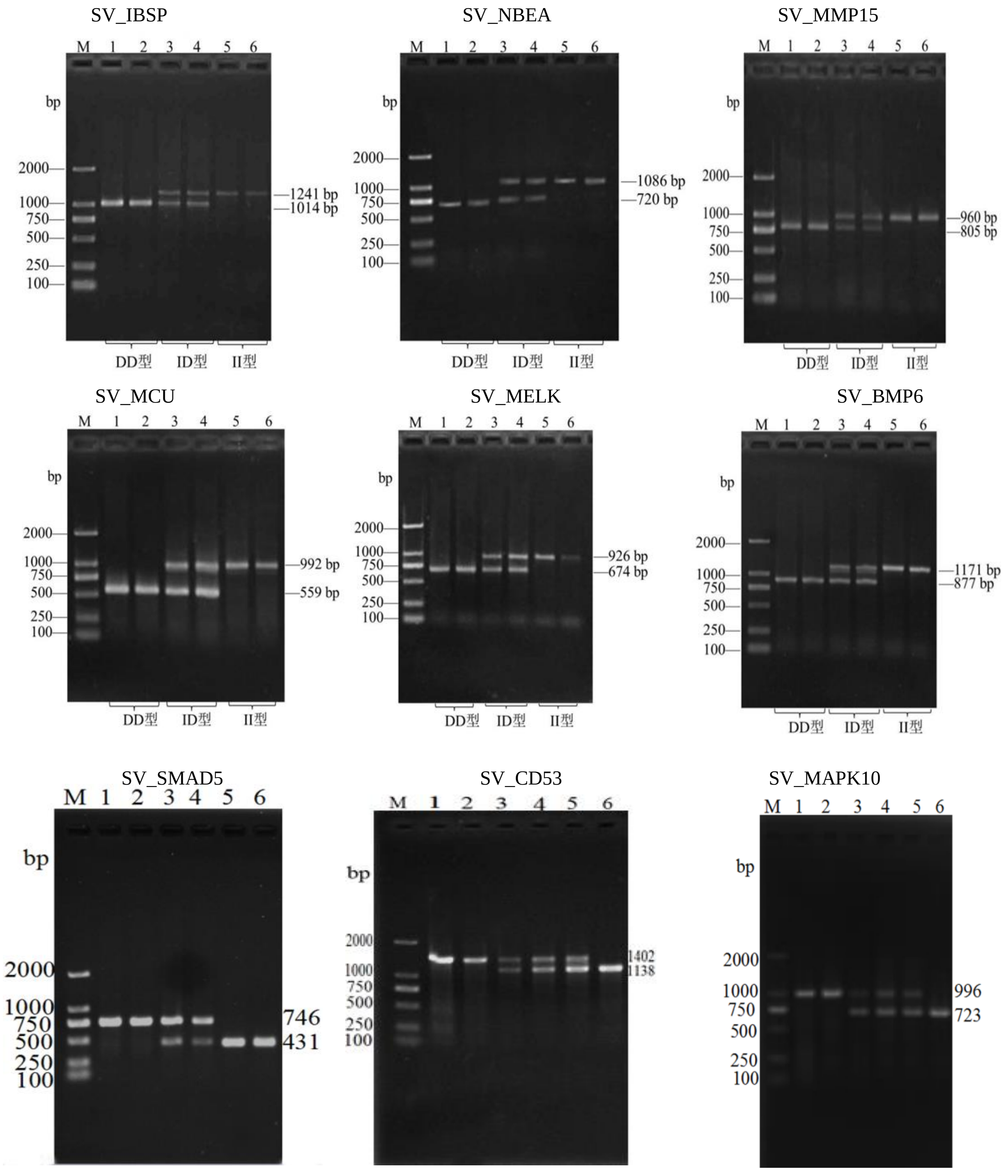

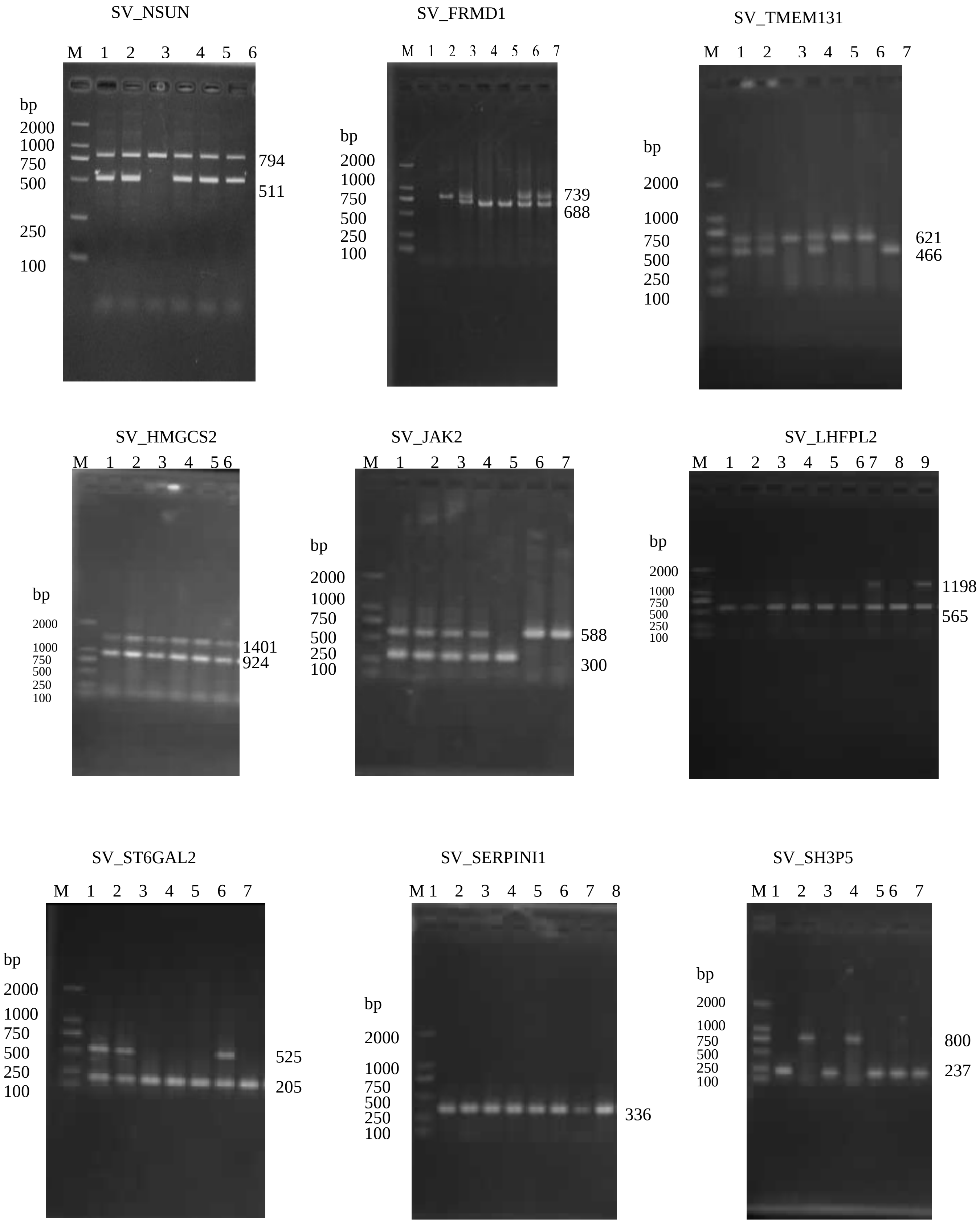
SV_NSUN
M 1 2 3 4 5 6
bp
2000
1000
750
500
250
100
794
511
SV_FRMD1
M 1 2 3 4 5 6 7
bp
2000
1000
750
500
250
100
739
688
SV_TMEM131
M 1 2 3 4 5 6 7
bp
2000
1000
750
500
250
100
621
466
SV_HMGCS2
M 1 2 3 4 5 6
bp
2000
1000
750
500
250
100
1401
924
SV_JAK2
M 1 2 3 4 5 6 7
bp
2000
1000
750
500
250
100
588
300
SV_LHFPL2
M 1 2 3 4 5 6 7 8 9
bp
2000
1000
750
500
250
100
1198
565
SV_ST6GAL2
M 1 2 3 4 5 6 7
bp
2000
1000
750
500
250
100
525
205
SV_SERPINI1
M 1 2 3 4 5 6 7 8
bp
2000
1000
750
500
250
100
336
SV_SH3P5
M 1 2 3 4 5 6 7
bp
2000
1000
750
500
250
100
800
237

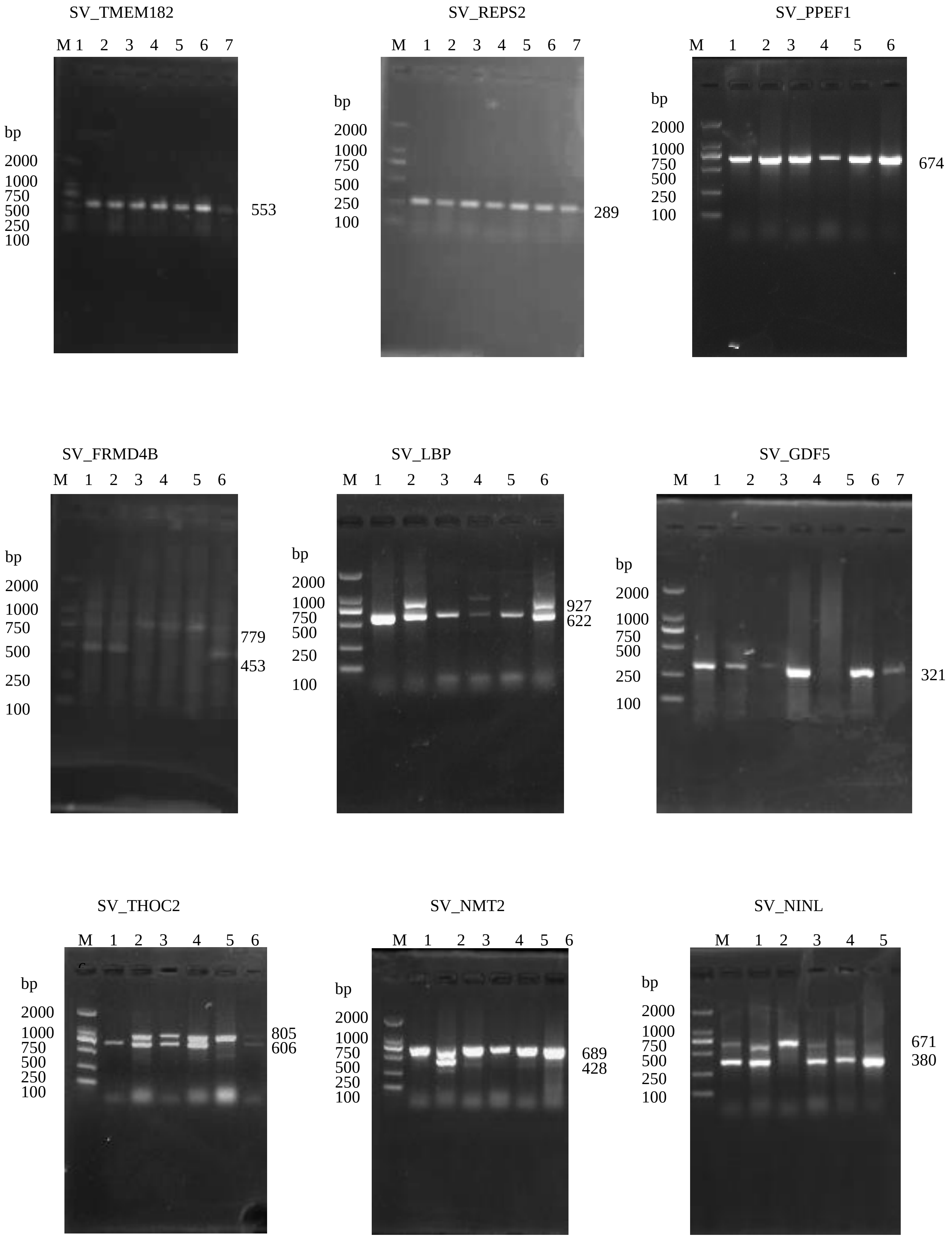
SV_TMEM182
M 1 2 3 4 5 6 7
bp
2000
1000
750
500
250
100
553
SV_REPS2
M 1 2 3 4 5 6 7
bp
2000
1000
750
500
250
100
289
SV_PPEF1
M 1 2 3 4 5 6
bp
2000
1000
750
500
250
100
674
SV_FRMD4B
M 1 2 3 4 5 6
bp
2000
1000
750
500
250
100
779
453
SV_LBP
M 1 2 3 4 5 6
bp
2000
1000
750
500
250
100
927
622
SV_GDF5
M 1 2 3 4 5 6 7
bp
2000
1000
750
500
250
100
321
SV_THOC2
M 1 2 3 4 5 6
bp
2000
1000
750
500
250
100
805
606
SV_NMT2
M 1 2 3 4 5 6
bp
2000
1000
750
500
250
100
689
428
SV_NINL
M 1 2 3 4 5
bp
2000
1000
750
500
250
100
671
380

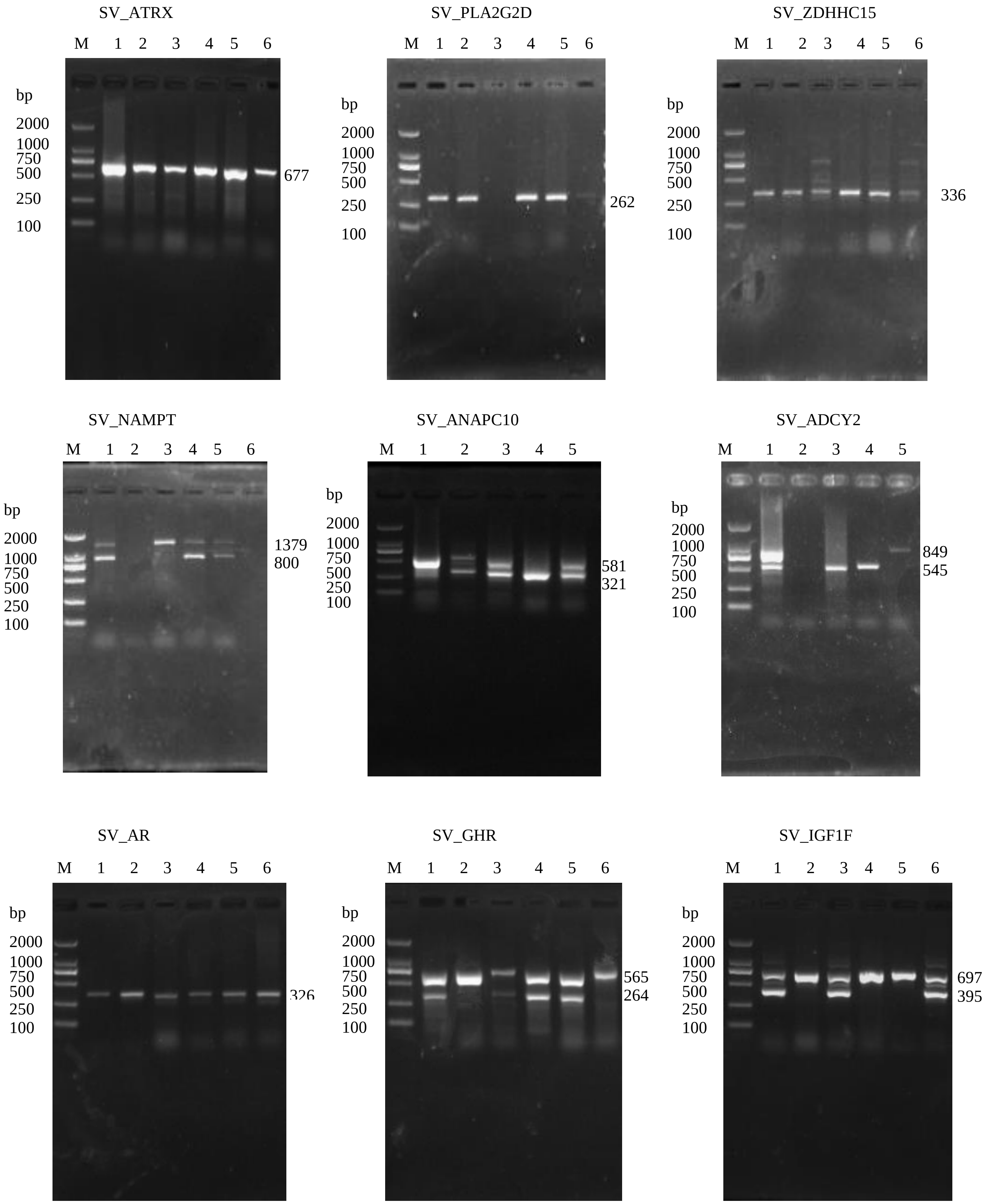

SV_ATRX
M 1 2 3 4 5 6
bp
2000
1000
750
500
250
100
677
SV_PLA2G2D
M 1 2 3 4 5 6
bp
2000
1000
750
500
250
100
262
SV_ZDHHC15
M 1 2 3 4 5 6
bp
2000
1000
750
500
250
100
336
SV_NAMPT
M 1 2 3 4 5 6
bp
2000
1000
750
500
250
100
1379
800
SV_ANAPC10
M 1 2 3 4 5
bp
2000
1000
750
500
250
100
581
321
SV_ADCY2
M 1 2 3 4 5
bp
2000
1000
750
500
250
100
849
545
SV_AR
M 1 2 3 4 5 6
bp
2000
1000
750
500
250
100
326
SV_GHR
M 1 2 3 4 5 6
bp
2000
1000
750
500
250
100
565
264
SV_IGF1F
M 1 2 3 4 5 6
bp
2000
1000
750
500
250
100
697
395

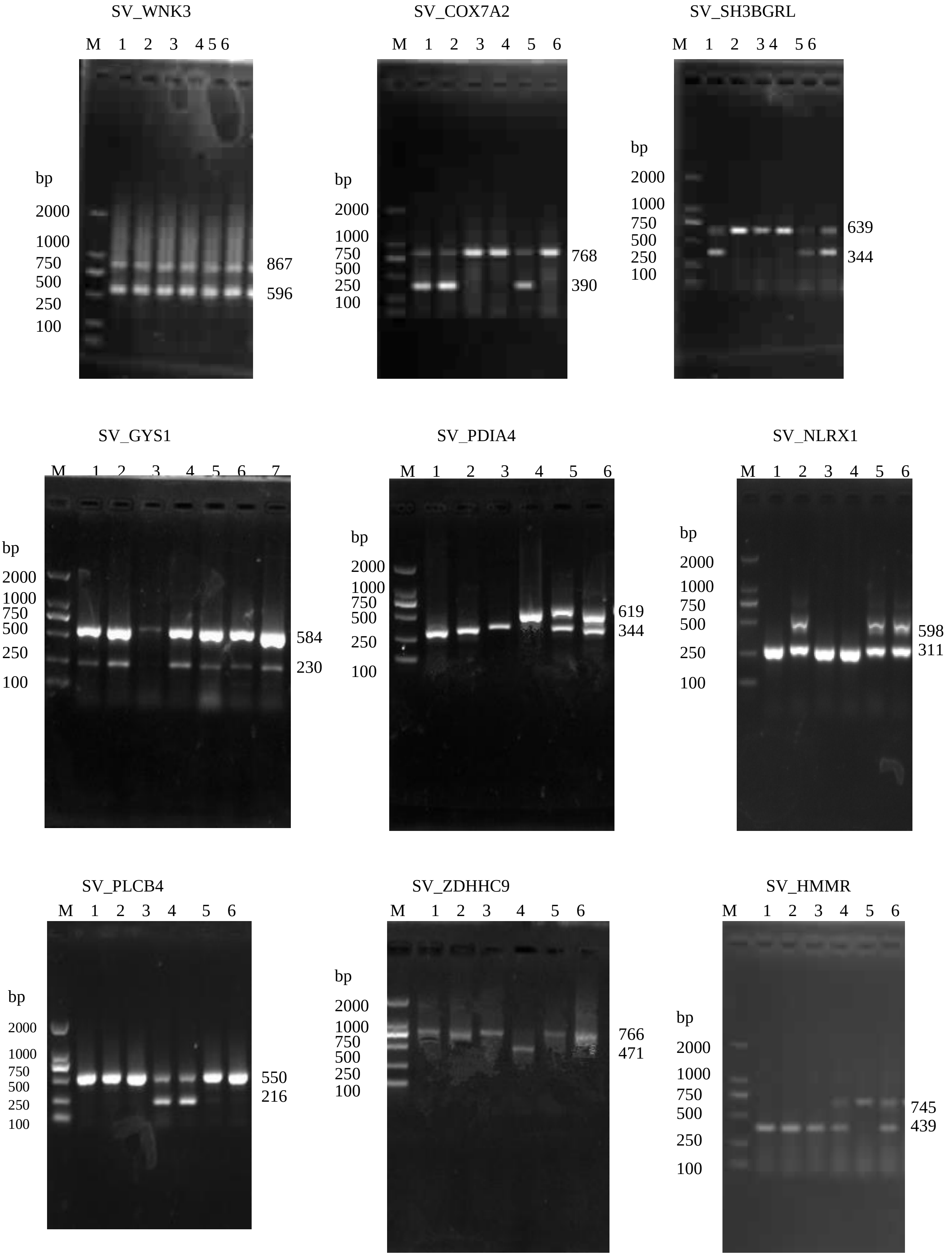
SV_WNK3
M 1 2 3 4 5 6
bp
2000
1000
750
500
250
100
867
596
SV_COX7A2
M 1 2 3 4 5 6
bp
2000
1000
750
500
250
100
768
390
SV_SH3BGRL
M 1 2 3 4 5 6
bp
2000
1000
750
500
250
100
639
344
SV_GYS1
M 1 2 3 4 5 6 7
bp
2000
1000
750
500
250
100
584
230
SV_PDIA4
M 1 2 3 4 5 6
bp
2000
1000
750
500
250
100
619
344
SV_NLRX1
M 1 2 3 4 5 6
bp
2000
1000
750
500
250
100
598
311
SV_PLCB4
M 1 2 3 4 5 6
bp
2000
1000
750
500
250
100
550
216
SV_ZDHHC9
M 1 2 3 4 5 6
bp
2000
1000
750
500
250
100
766
471
SV_HMMR
M 1 2 3 4 5 6
bp
2000
1000
750
500
250
100
745
439

SV_ITGB3

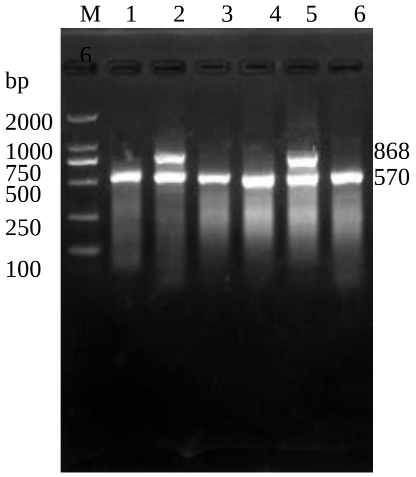

SV_PRKAA2

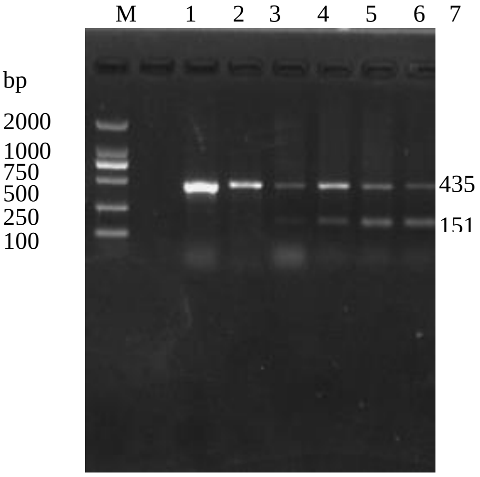

SV_LEXM

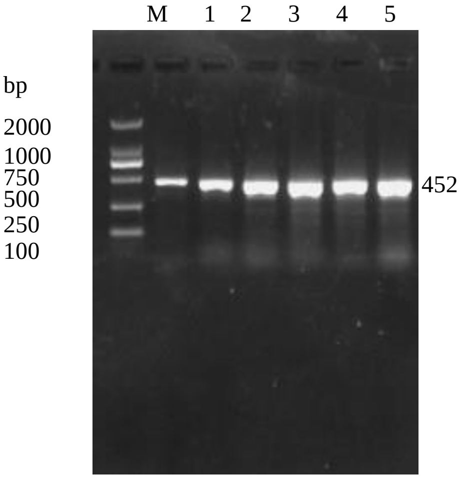

SV_ARNT

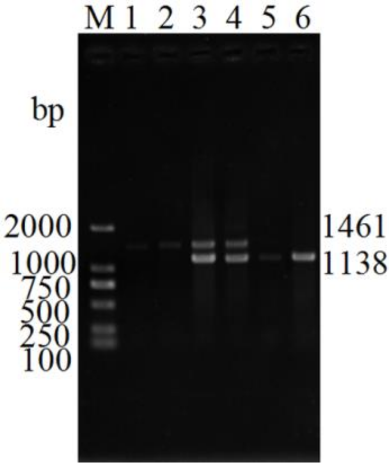

SV_COL6A5

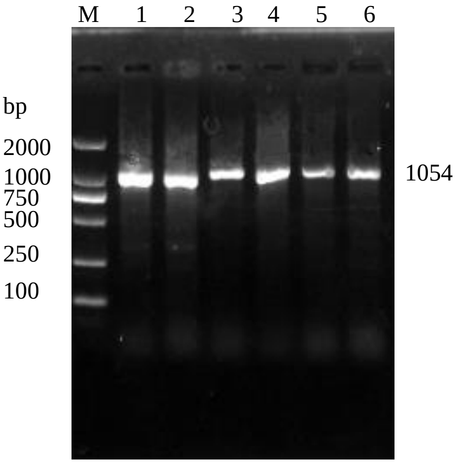

SV_ADH5

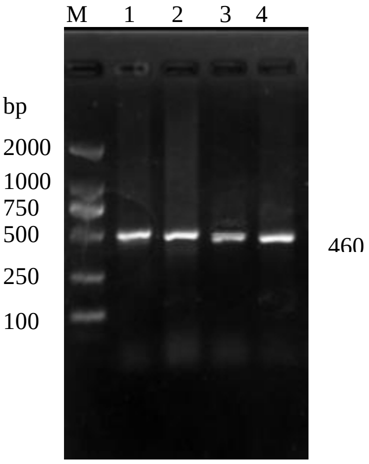

SV_HSD17B12

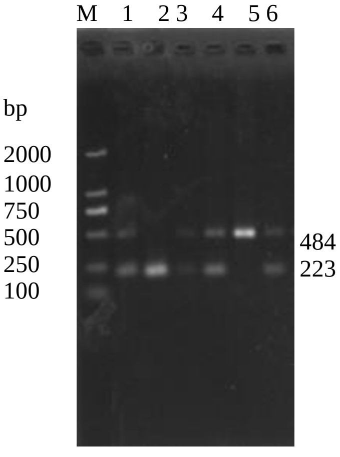

SV_GRIA3

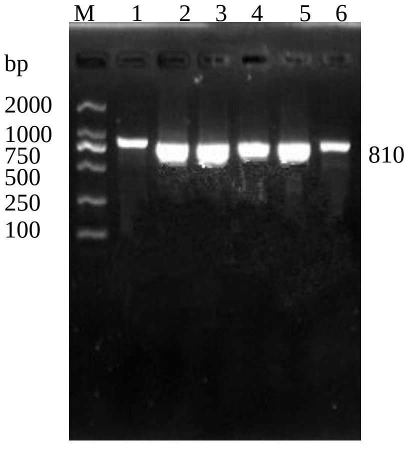

SV_FAM49A

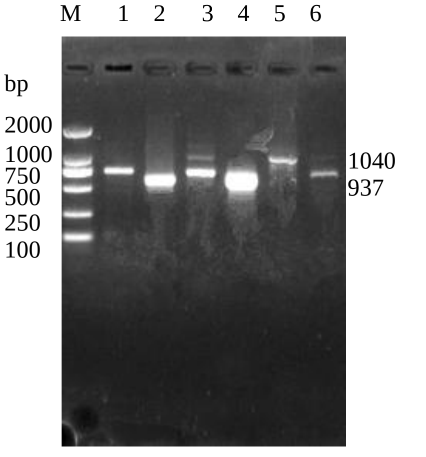

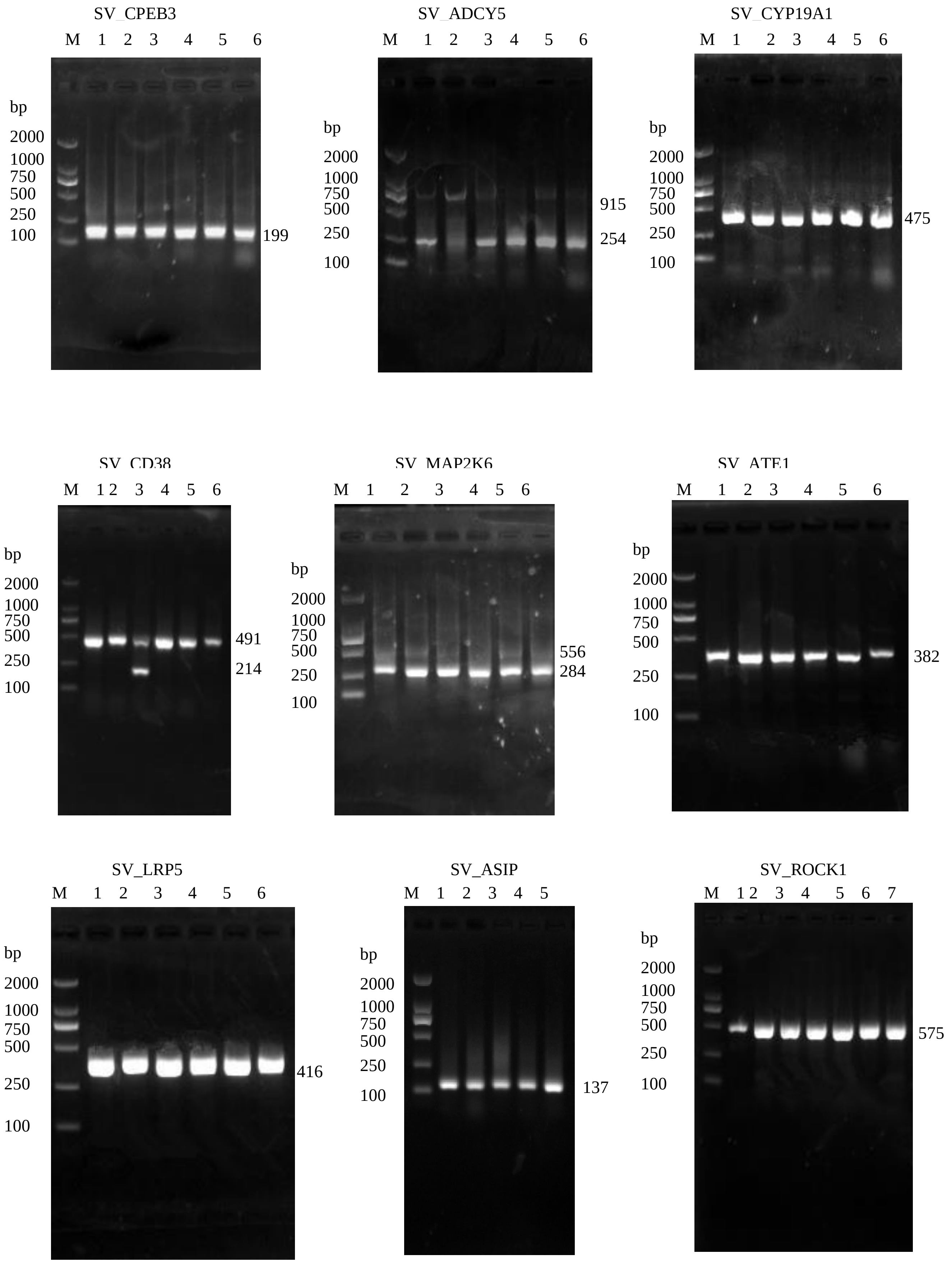
SV_CPEB3
M 1 2 3 4 5 6
bp
2000
1000
750
500
250
100
199
SV_ADCY5
M 1 2 3 4 5 6
bp
2000
1000
750
500
250
100
915
254
SV_CYP19A1
M 1 2 3 4 5 6
bp
2000
1000
750
500
250
100
475
M 1 2 3 4 5 6
bp
2000
1000
750
500
250
100
491
214
M 1 2 3 4 5 6
bp
2000
1000
750
500
250
100
556
284
M 1 2 3 4 5 6
bp
2000
1000
750
500
250
100
382
SV_LRP5
M 1 2 3 4 5 6
bp
2000
1000
750
500
250
100
416
SV_ASIP
M 1 2 3 4 5
bp
2000
1000
750
500
250
100
137
SV_ROCK1
M 1 2 3 4 5 6 7
bp
2000
1000
750
500
250
100
575

SV_RSPO2

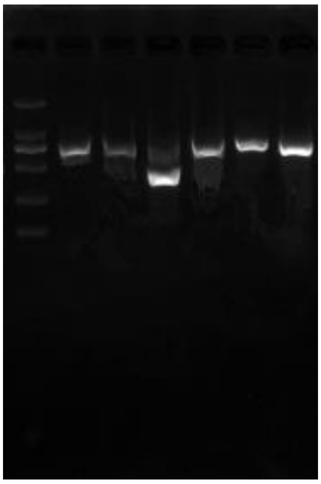

SV_KLF3

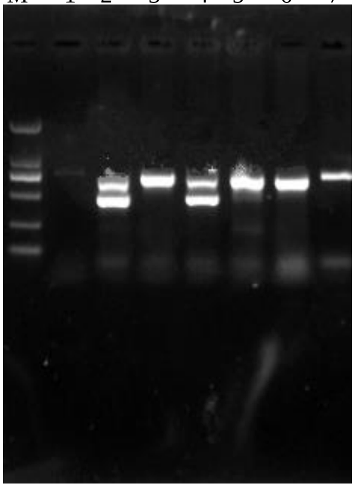

SV_WNT2

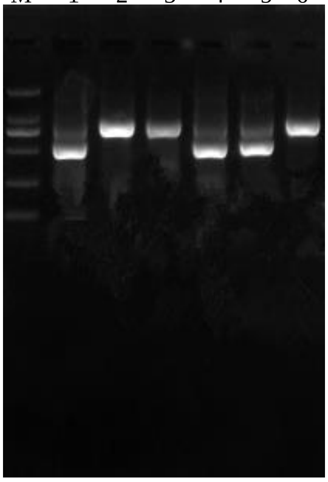

SV_ACAT2

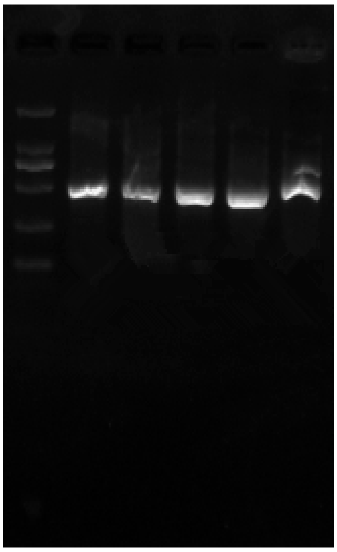

SV_ACSL3

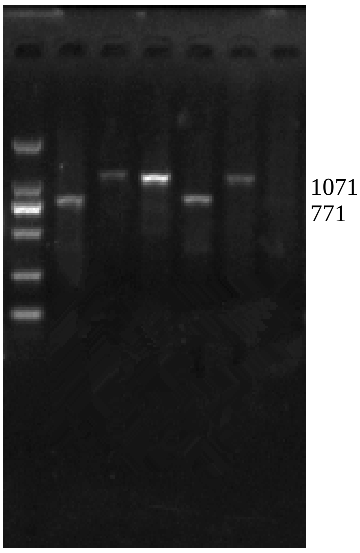

SV_HADH

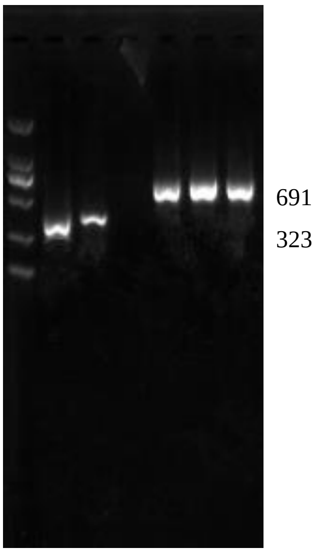

SV_APOD

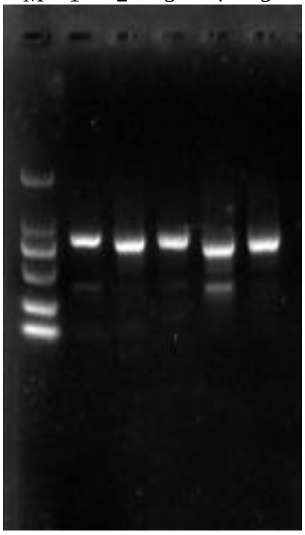

SV_GSK3B

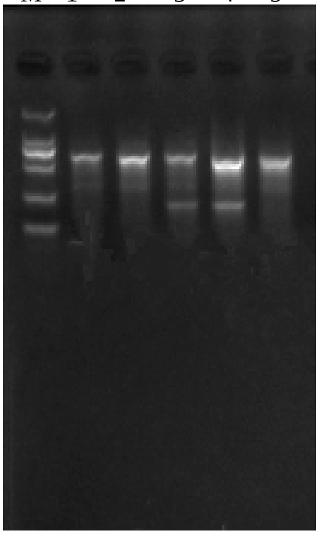

SV_AKT3

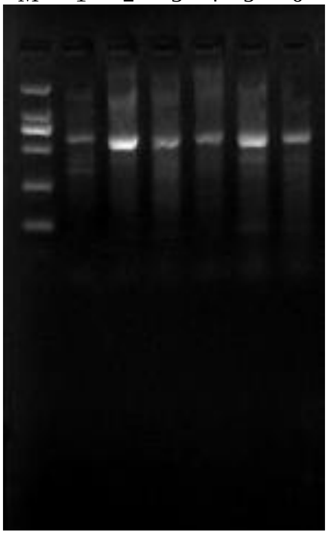

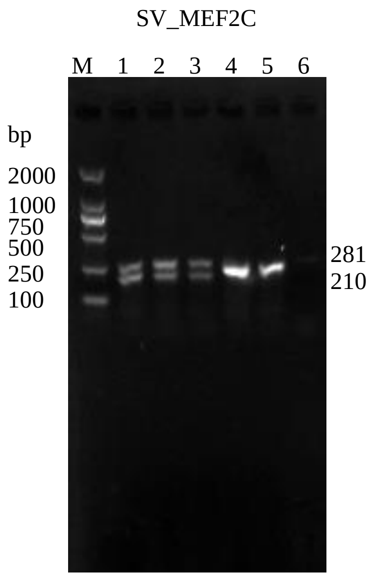
SV_MEF2C
M 1 2 3 4 5 6
bp
2000
1000
750
500
250
100
281
210

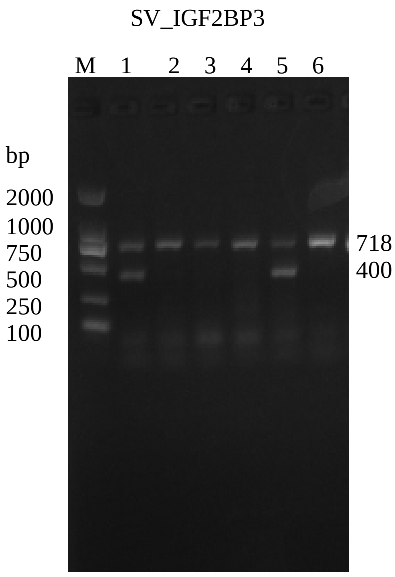
SV_IGF2BP3
M 1 2 3 4 5 6
bp
2000
1000
750
500
250
100
718
400

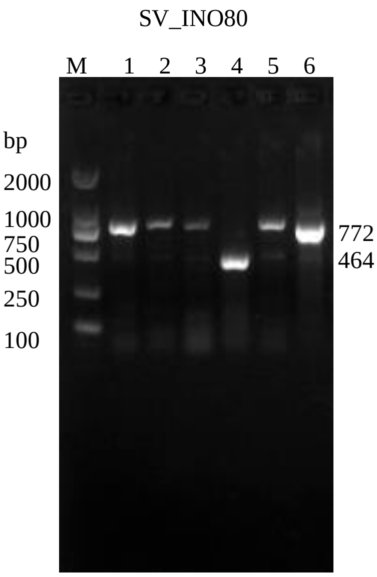
SV_INO80
M 1 2 3 4 5 6
bp
2000
1000
750
500
250
100
772
464

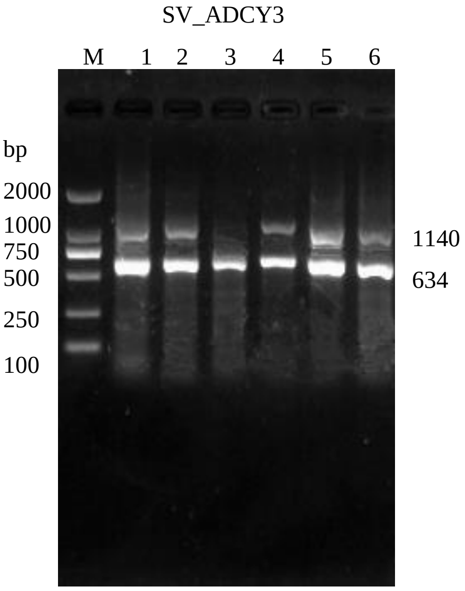
SV_ADCY3
M 1 2 3 4 5 6
bp
2000
1000
750
500
250
100
1140
634
